# A Hypothalamic Oxytocinergic–Sympathetic Axis Couples Anxiety Dysregulation With Impaired Tendon–Bone Repair

**DOI:** 10.1002/advs.78190

**Published:** 2026-10-06

**Authors:** Liyang Wan, Tingmo Huang, Liyue Zeng, Ruixue Du, Hengshuo Zhang, Shen Liu, Tao Zhang, Jianzhong Hu, Hongbin Lu

**Affiliations:** ^1^ Department of Sports Medicine, Xiangya Hospital Central South University Changsha China; ^2^ Key Laboratory of Organ Injury Aging and Regenerative Medicine of Hunan Province Changsha China; ^3^ National Clinical Research Center for Geriatric Disorders, Xiangya Hospital Central South University Changsha China; ^4^ Department of Orthopaedics Xiangya Hospital Central South University Changsha China

**Keywords:** anxiety, Functional Magnetic Resonance Imaging (fMRI), hypothalamus, oxytocinergic neurons, paraventricular nucleus, rotator cuff injury

## Abstract

Rotator cuff injury (RCI) is frequently accompanied by psychological comorbidities, particularly anxiety‐related symptoms, which predict poor functional recovery, yet the central mechanisms linking affective disturbance to impaired tendon–bone healing remain unclear. Here, by integrating clinical neuroimaging, imaging transcriptomics, and mechanistic animal experiments, we identify hypothalamic paraventricular nucleus (PVN) oxytocinergic (OXT) neurons as a critical central node coordinating emotional regulation and bone–tendon interface (BTI) repair. In patients with RCI, resting‐state fMRI (rs‐fMRI) revealed reduced hypothalamic spontaneous activity that was inversely correlated with anxiety severity. Imaging transcriptomic analyses further linked these functional alterations to spatially concordant gene expression programs enriched in oxytocin signaling. In mouse models, retrograde viral tracing and spatial transcriptomics identified PVN^OXT^ neurons as the predominant labeled population within a polysynaptic PVN–IML–SCG sympathetic pathway connected to the subchondral bone region of the rotator cuff enthesis. In vivo local field potential recordings confirmed a reduction in PVN neuronal activity that peaked at four weeks post‐injury and recovered thereafter, paralleling the behavioral time course. Chemogenetic inhibition of PVN^OXT^ neurons induced anxiety‐like behaviors and impaired BTI healing, whereas their activation alleviated anxiety and enhanced structural and biomechanical recovery. Together, these findings support a working model in which a PVN^OXT–^sympathetic‐shoulder axis links anxiety dysregulation to musculoskeletal healing through a hypothalamic–sympathetic pathway, providing a framework for integrated neuromodulatory and tissue‐targeted interventions in RCI.

## Graphical abstract

RCI is associated with functional suppression of oxytocinergic neurons in the PVN. Reduced PVN^OXT^ neuronal activity contributes to altered sympathetic regulation involving the IML–sympathetic chain pathway, accompanied by changes in norepinephrine signaling at the BTI. This PVN‐associated sympathetic activation is accompanied by increased anxiety‐like behavior and impaired structural and biomechanical healing of the BTI, identifying a PVN^OXT^–sympathetic–shoulder axis that may contribute to the emotional disturbance with musculoskeletal repair.

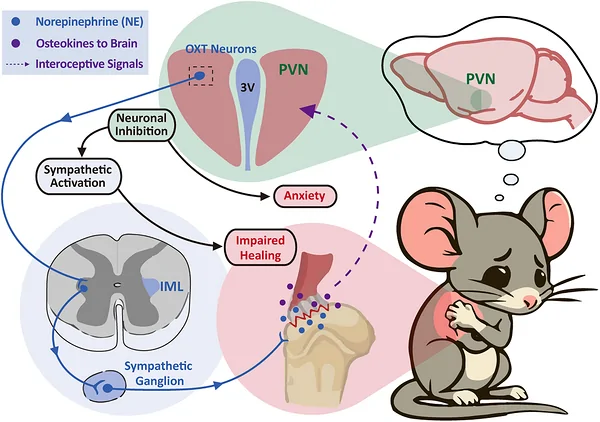

## 1 Introduction

Rotator cuff injury (RCI) is the most prevalent disorder of the upper extremity and a leading cause of shoulder pain and disability worldwide [1]. Beyond pain and functional limitation, a substantial proportion of patients with RCI experience significant psychological comorbidities, most prominently anxiety, which independently predict inferior postoperative outcomes following rotator cuff repair [2, 3, 4, 5]. Although these psychological comorbidities are increasingly recognized as clinically relevant, they are often regarded as secondary consequences of pain and disability, and the central mechanisms through which they interact with tissue repair remain poorly understood.

Accumulating evidence suggests that musculoskeletal injury can induce plastic changes in the central nervous system (CNS), which in turn modulate peripheral tissue homeostasis through descending autonomic pathways [6]. Among these, the sympathetic nervous system plays a pivotal role in bone and tendon biology, including regulation of osteogenesis, angiogenesis, and inflammatory responses at the bone–tendon interface (BTI) [6, 7, 8]. Experimental manipulation of peripheral sympathetic tone has been shown to markedly influence BTI healing quality, highlighting the importance of central control over autonomic output in musculoskeletal repair [7, 9, 10, 11]. However, the CNS components that integrate emotional processing with autonomic output to influence BTI healing have not been systematically defined.

The hypothalamus is uniquely positioned to serve as such an integrative hub. As a core regulator of emotional behavior and autonomic function, the hypothalamus coordinates affective states with peripheral physiological responses [12]. Anxiety‐related behavior and stress‐evoked autonomic output are governed by a distributed hypothalamic–brainstem network that includes the corticotropin‐releasing hormone (CRH)–hypothalamic–pituitary–adrenal axis, vasopressinergic circuits, the locus coeruleus–noradrenergic system, and oxytocinergic circuits, each of which could in principle couple affective disturbance to peripheral physiology. Which, if any, of these systems is engaged in RCI has not been examined. Within the hypothalamus, the paraventricular nucleus (PVN) contains oxytocinergic (OXT) neurons that participate in anxiety regulation and project to autonomic centers controlling sympathetic outflow [13, 14, 15, 16], suggesting that PVN^OXT^ neurons may represent a convergence point between emotional regulation and autonomic mechanisms relevant to musculoskeletal repair. Nevertheless, whether dysfunction of this neuronal population contributes to anxiety‐associated impairment of BTI healing in RCI has not been directly examined.

In the present study, we adopted a translational, multiscale strategy to address this gap. First, using resting‐state functional MRI (rs‐fMRI) in patients with RCI, we identified hypothalamus‐centered functional alterations associated with anxiety severity. Second, by integrating neuroimaging findings with imaging transcriptomics, we explored molecular pathways spatially associated with hypothalamic dysfunction, with particular emphasis on oxytocin signaling. Third, employing retrograde viral tracing, spatial transcriptomics, and chemogenetic manipulation in mouse models, we delineated a PVN‐centered sympathetic circuit projecting to the rotator cuff enthesis subchondral area and provided evidence that PVN^OXT^ neuronal activity contributes to regulating anxiety‐like behavior and BTI healing. Together, these approaches enabled us to define a brain–body pathway through which emotional and musculoskeletal pathologies are mechanistically coupled in RCI.

## 2 Results

### 2.1 Anxiety as the Dominant Psychological Phenotype in RCI is Associated With Suppressed Hypothalamic Activity and Spatially Concordant Oxytocin Signaling

The overall study workflow is summarized in Figure 1A. Clinical scale assessments revealed that patients with RCI exhibited markedly higher prevalence and severity of anxiety compared with healthy controls (HADS‐A: RCI severe/moderate/mild proportion = 27.9% vs. controls = 10%; Mann–Whitney U = 892.5, *p* = 0.0053), whereas depression levels did not differ significantly between groups (HADS‐D: Mann–Whitney U = 759.0, *p* = 0.1992) (Figure 1B and Table S1). This dissociation indicates that anxiety, rather than depression, represents the predominant psychological phenotype in RCI.

**FIGURE 1:**
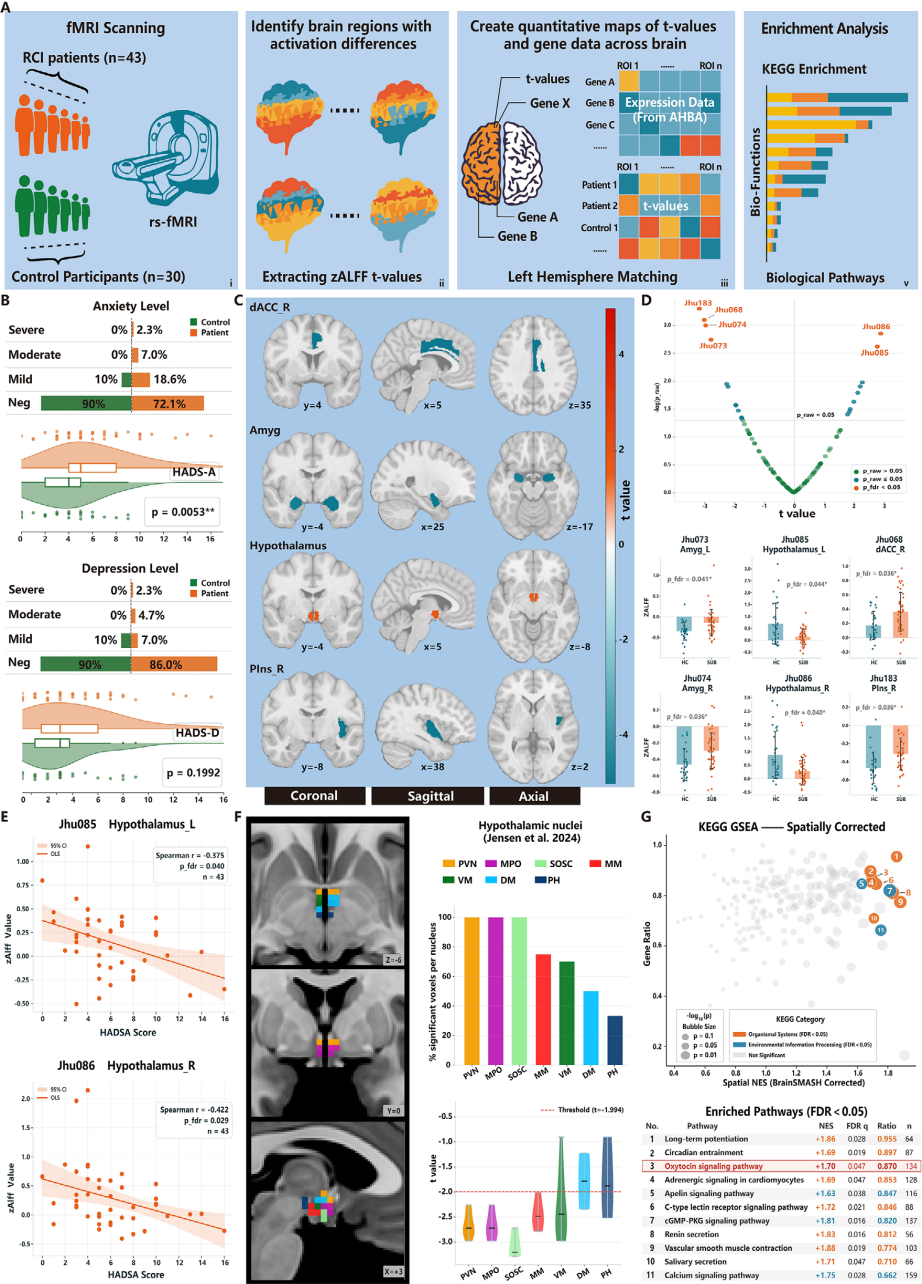
Hypothalamus‐centered resting‐state functional alterations and spatially concordant molecular signatures in patients with RCI. (A) Study workflow. (i) rs‐fMRI was acquired from patients with RCI (n = 43) and healthy controls (n = 30). (ii) Whole‐brain z‐transformed amplitude of low‐frequency fluctuations (zALFF) was computed. (iii) Two‐sample group comparisons generated region‐level t‐statistic maps, which were spatially matched to gene expression data from the Allen Human Brain Atlas (AHBA) across the left‐hemisphere ROIs defined by the JHU atlas. (iv) KEGG pathway enrichment analysis was performed based on region–gene expression associations, with spatial correction applied via BrainSMASH permutation testing. (B) Distribution of anxiety (HADS‐A, upper) and depression (HADS‐D, lower) scores in patients with RCI and healthy controls. Stacked bar plots show proportions of each severity category (Negative, Mild, Moderate, Severe). Raincloud plots display individual score distributions with embedded boxplots. RCI patients exhibited significantly elevated anxiety scores (Mann–Whitney U = 892.5, *p* = 0.0053), whereas depression scores did not differ significantly between groups (Mann–Whitney U = 759.0, *p* = 0.1992). Stacked bars are descriptive; group comparisons were made on continuous scores by Mann–Whitney U test. n = 43 patients, 30 controls. (C) Statistical parametric maps (T‐maps) displaying brain regions with significantly altered zALFF in RCI patients relative to controls, shown in coronal, sagittal, and axial views. Warm colors (positive t‐values, contrast: HC − Patient) indicate regions where spontaneous activity is reduced in patients relative to controls, observed bilaterally in the hypothalamus. Cool colors (negative t‐values) indicate regions where activity is relatively increased in patients, including the right dACC, bilateral amygdala, and right posterior insula. Two‐sample *t*‐tests with age and sex as covariates, two‐tailed. (D) Volcano plot of ROI‐level zALFF group differences. Each dot represents one gray‐matter ROI defined by the JHU atlas. Blue dots indicate ROIs reaching uncorrected significance (p_raw < 0.05); orange dots indicate ROIs surviving FDR correction. Six key regions are annotated: JHU073 (Amyg_L), JHU074 (Amyg_R), JHU068 (dACC_R), JHU183 (PIns_R), JHU085 (Hypothalamus_L), and JHU086 (Hypothalamus_R). Adjacent box plots display extracted zALFF values for healthy controls (HC) and RCI patients (SUB) within each annotated ROI, with corresponding FDR‐corrected *p*‐values indicated. (E) Scatter plots illustrating the association between hypothalamic zALFF values and individual HADS‐A score across RCI patients (n = 43). Upper: left hypothalamus (JHU085; Spearman r = −0.375, P_FDR = 0.040). Lower: right hypothalamus (JHU086; Spearman r = −0.422, P_FDR = 0.029). Solid lines represent OLS regression fits; shaded areas denote 95% confidence intervals. No significant correlations were observed between HADS‐A scores and zALFF values of the remaining four FDR‐significant regions. (F) Subnuclei‐level dissection of hypothalamic zALFF alterations using the Jensen et al. [18] human hypothalamic atlas. Left column: MRI sections at three anatomical levels (Z = −6, Y = 0, X = 3) with color‐coded delineation of seven functional subnuclei (PVN, MPO, SOSC, MM, VM, DM, PH). Upper right: bar graph showing the percentage of voxels per subnucleus surpassing the statistical threshold; PVN, MPO, and SOSC exhibit substantially higher proportions than the remaining subnuclei. Lower right: violin plots of subnucleus‐level t‐value distributions; the dashed horizontal line marks the threshold t = −1.994. PVN, MPO, and SOSC distributions are predominantly below this threshold, indicating consistent and localized functional suppression, whereas other subnuclei show negligible contributions. (G) Spatially corrected KEGG gene set enrichment analysis (GSEA) integrating hypothalamic zALFF differential maps with AHBA gene expression data. Main scatter plot: each dot represents one KEGG pathway; the *x*‐axis shows the spatially corrected normalized enrichment score (Spatial NES, BrainSMASH‐corrected) and the y‐axis shows the gene ratio. Dot size reflects −log_10_(p); orange dots denote significantly enriched organismal systems pathways (P_FDR_ < 0.05) and blue dots denote significantly enriched environmental information processing pathways (P_FDR_ < 0.05). Inset table: pathways surviving FDR correction (q < 0.05), ranked by gene ratio. The oxytocin signaling pathway (highlighted in red, No. 3; NES = +1.70, FDR q = 0.047, n = 134 genes) represents one of the most biologically relevant enriched pathways spatially concordant with hypothalamic functional suppression in RCI.

To determine whether RCI is accompanied by detectable alterations in central nervous system spontaneous activity, rs‐fMRI was performed in patients (n = 43) and healthy controls (n = 30). Whole‐brain parcellation‐based analysis using the JHU atlas based on z‐transformed amplitude of low‐frequency fluctuations (zALFF) identified six gray‐matter regions (JHU068, 073, 074, 085, 086, 183) exhibiting significantly altered spontaneous activity, including the right dorsal anterior cingulate cortex (dACC_R), bilateral amygdala, bilateral hypothalamus, and right posterior insula (PIns_R) (Figure 1C,D and Table S2) [17]. Notably, whereas the dACC_R, bilateral amygdala, and PIns_R showed increased zALFF in patients, both hypothalamic regions (JHU085 and JHU086) demonstrated significantly reduced zALFF values (P_FDR_ = 0.044 and 0.040, respectively) (Figure 1D). To explore whether the altered activity across these regions was associated with anxiety severity, Spearman correlation analyses were performed between regional zALFF values and individual HADS‐A scores. Among all six regions exhibiting significant group differences, both the left and right hypothalamic zALFF values showed a significant inverse correlation with anxiety severity (left: r = −0.375, P_FDR = 0.040; right: r = −0.422, P_FDR = 0.029; n = 43) (Figure 1E), indicating that greater hypothalamic suppression corresponds to more severe anxiety symptoms (Table S3). We note that these associations are not fully subscale‐specific: HADS‐D correlations within the same regions were of comparable magnitude (left r = −0.388; right r = −0.315) and failed FDR correction only after separate within‐subscale adjustment (Table S3). The anxiety‐dominance of this cohort is therefore supported principally at the group level, where HADS‐A differed between patients and controls whereas HADS‐D did not. These results implicate hypothalamic functional suppression, rather than the broader pattern of altered spontaneous activity, as the central neural correlate most closely tracking affective symptom severity in RCI.

Given the pronounced functional heterogeneity among hypothalamic subnuclei, we sought to identify which specific subnuclear compartments contributed most to the observed hypothalamic suppression. To this end, we leveraged the hypothalamic atlas proposed by Jensen et al. to map the distribution of zALFF reductions across seven functional subnuclei (Figure 1F) [18]. Among these, the paraventricular nucleus (PVN), medial preoptic nucleus (MPO), and supraoptic nucleus superior compartment (SOSC) contained the highest proportions of voxels surpassing the statistical threshold (|t| > 1.994) (Figure 1F).

Finally, to identify molecular pathways spatially concordant with central functional alterations, imaging transcriptomic analyses were conducted by integrating the hypothalamic zALFF differential maps with gene expression data from the Allen Human Brain Atlas (AHBA) [19]. Spatially corrected KEGG gene set enrichment analysis (GSEA) identified the oxytocin signaling pathway as a significant hit (NES = +1.70, FDR q = 0.047), ranking among the top enriched pathways (Figure 1G). This result spatially links hypothalamic functional suppression to dysregulation of the oxytocinergic molecular program, providing a mechanistic rationale for subsequent circuit‐level investigations.

### 2.2 RCI Recapitulates Hypothalamic PVN Suppression and Anxiety‐Like Behavior in Mice With Temporally Coupled Sympathetic Activation, With Connectome Analysis Identifying PVN^OXT^ Neurons as a Candidate Integrative Node

To validate the translational relevance of the clinical findings, mice were subjected to RCI and assessed across multiple post‐injury time points using standardized behavioral paradigms. The results showed that RCI induced a time‐dependent anxiety‐like phenotype that peaked at four weeks post‐injury (4wks pi): compared with uninjured controls, mice at 4wks pi exhibited significantly reduced total locomotor distance and time spent in the center zone in the open field test (OFT), as well as markedly decreased open‐arm dwell time and entry frequency in the elevated plus maze (EPM) (all *p* < 0.001) (Figure 2A,B). Behavioral changes at 2wks and 8wks pi were substantially less pronounced, indicating that the subacute phase represents the peak time window of affective disturbance. These findings recapitulate the anxiety‐dominant phenotype observed in clinical patients and establish 4wks pi as the primary experimental time point.

**FIGURE 2:**
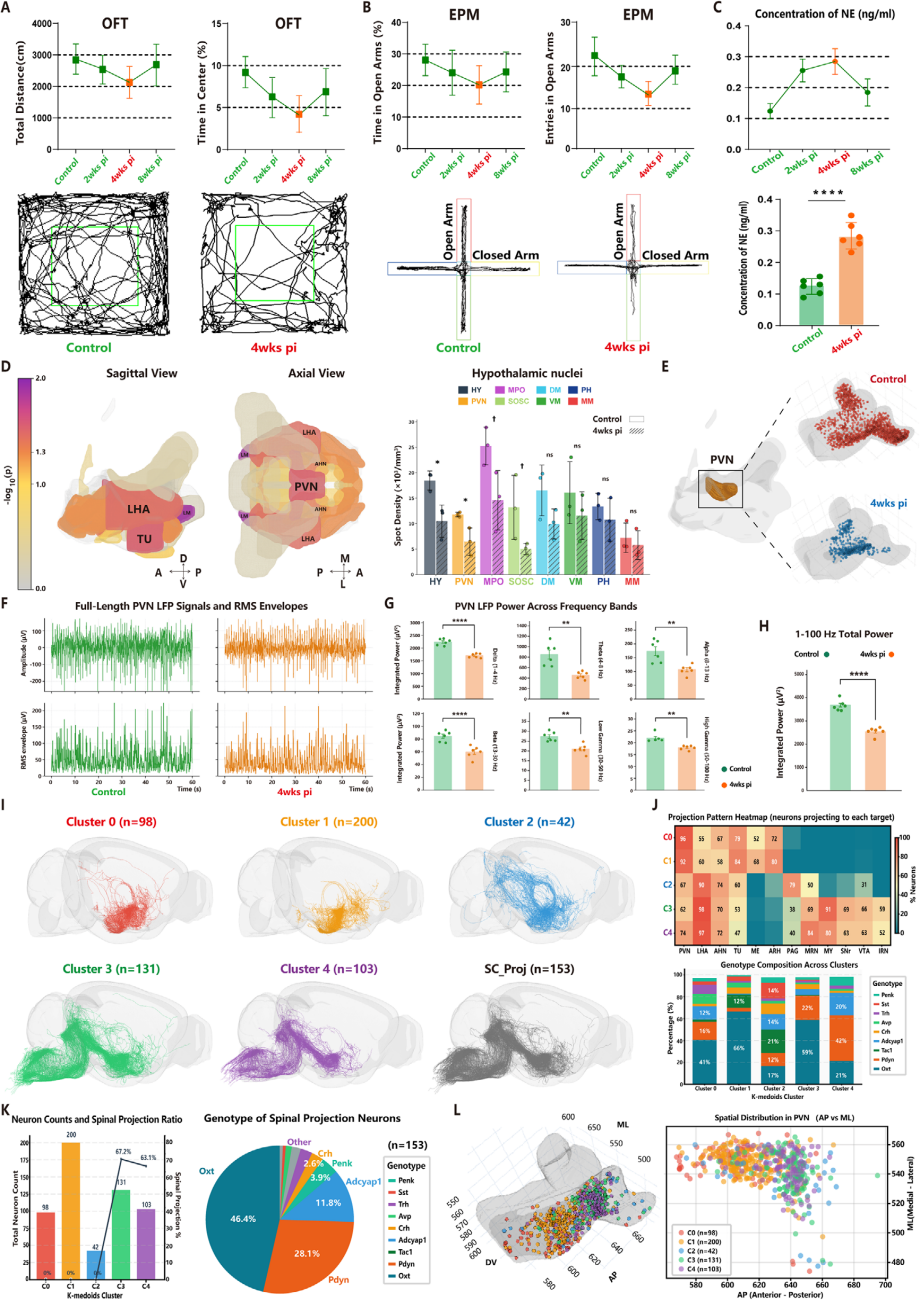
Suppressed PVN neuronal activity, anxiety‐like behavior, and peripheral sympathetic hyperactivation emerge as temporally coupled responses to RCI in mice, and connectome reanalysis identifies PVN^OXT^ neurons as a candidate integrative node. (A) Open field test (OFT) results across injury time points. Upper panels: group‐level total locomotor distance (cm, left) and percentage of time spent in the center zone (%, right) at Control, 2wks pi, 4wks pi, and 8wks pi (mean ± SEM). The 4wks pi group (orange) showed the most pronounced reductions in both parameters. Lower panels: representative locomotor tracking paths for Control (left) and 4wks pi (right) mice; green traces indicate center‐zone occupancy. n = 18 independently tested mice per time point; one‐way ANOVA test. The same n and test apply to (B). (B) Elevated plus maze (EPM) results across injury time points. Upper panels: percentage of time spent in open arms (%, left) and number of open‐arm entries (right) at each time point (mean ± SEM). 4wks pi mice showed the most severe anxiety‐like phenotype. Lower panels: representative arm‐occupancy trajectory plots for Control (left) and 4wks pi (right) mice. Each animal completed the open field test and elevated plus maze once only, at its assigned time point; no animal was tested more than once. (C) NE concentration at the BTI across time points. Upper: time‐course line graph showing NE levels (ng/mL, mean ± SEM) peaking at 4wks pi. Lower: comparison of NE concentration between Control and 4wks pi groups; individual data points shown as scatter overlay. n = 6 mice per time point; one‐way ANOVA test for the time course and Welch's two‐tailed *t*‐test for the Control vs. 4wks pi comparison. (D) Whole‐brain activity mapping using TRAP2 mice combined with optical clearing and light‐sheet fluorescence microscopy. Left: pseudocolor sagittal (left) and axial (right) views of the hypothalamic region displaying the spatial distribution of activated TRAP‐labeled (Fos‐driven tdTomato^+^) neurons; color scale represents −log_10_(p) values, with warmer colors indicating greater statistical significance of density differences. Key hypothalamic regions (PVN, LM, LHA, TU, AHN) are annotated. Right: quantitative comparison of TRAP‐labeled signal density (detected fluorescent foci per mm^3^, ×10^3^, mean ± SEM) between Control (solid bars) and 4wks pi (hatched bars) groups, shown for the hypothalamus as a whole (HY) and for the seven a priori functional subnuclei corresponding to the human hypothalamic atlas used in Figure 1F (PVN, MPO, SOSC, DM, VM, PH, MM). Among these subnuclei, the PVN showed the most significant reduction. Statistical significance indicated as ^*^ *p* < 0.05, †*p* < 0.1, ns = not significant. Statistics for all 28 hypothalamic nuclei surveyed are provided in Table S4. n = 3 mice per group; two‐tailed unpaired Student's *t*‐test. (E) Three‐dimensional reconstruction of activated PVN neurons from TRAP2 light‐sheet imaging. Control group (upper, red point cloud) and 4wks pi group (lower, blue point cloud) are shown against a transparent whole‐brain reference. Inset: anatomical location of the PVN within the full brain volume. The 4wks pi group exhibits markedly sparser Fos^TRAP^ labeling, with the reduction most pronounced in the medial and posterior PVN subregion. (F) Representative full‐length (60 s) in vivo PVN LFP traces (upper) and corresponding RMS envelopes (lower), recorded under isoflurane anesthesia from a Control (green) and a 4wks pi (orange) mouse. (G) Integrated LFP power across six canonical frequency bands — delta (1–4 Hz), theta (4–8 Hz), alpha (8–13 Hz), beta (13–30 Hz), low gamma (30–50 Hz), and high gamma (50–100 Hz) — in Control and 4wks pi groups. (H) Integrated 1–100 Hz broadband power. n = 6 mice per group. Data are presented as mean ± SEM with individual animals overlaid. Welch's two‐tailed *t*‐test with Benjamini–Hochberg FDR correction across bands. ^**^ *p* < 0.01, ^****^ *p* < 0.0001. The full post‐injury time course (Control, 2wks, 4wks and 8wks pi) is shown in Figure S1. (I) K‐medoids clustering of PVN projection neurons based on whole‐brain axonal projection patterns. Five distinct clusters are identified: Cluster 0 (n = 98, red), Cluster 1 (n = 200, orange), Cluster 2 (n = 42, blue), Cluster 3 (n = 131, green), and Cluster 4 (n = 103, purple). The spinal‐cord‐projecting subset is separately visualized (SC_Proj, n = 153, gray). Three‐dimensional morphological renderings are shown for each cluster against a transparent brain reference. (J) Upper: projection pattern heatmap showing the percentage of neurons in each cluster projecting to major downstream targets (PVN, LHA, AHN, TU, ME, ARH, PAG, MRN, MY, SNr, VTA, IRN); color intensity represents projection proportion (0%–100%). Cluster 3 exhibits particularly high PAG and brainstem projection ratios. Lower: stacked bar graphs showing the proportional genotype composition (based on neuropeptide marker expression: *Penk, Sst, Trh, Avp, Crh, Adcyap1, Tac1, Pdyn, Oxt*) across the five K‐medoids clusters. (K) Left: bar graph displaying total neuron counts per cluster (left *y*‐axis) alongside the proportion of neurons within each cluster that project to the spinal cord (right *y*‐axis, line plot). Cluster 2 contributes no spinal‐projecting neurons (0%), whereas Cluster 3 and Cluster 4 contribute 67.2% and 63.1%, respectively. Right: pie chart showing the neuropeptide genotype composition of the 153 spinal‐projecting neurons (SC_Proj); *Oxt* constitutes the largest single genotype (46.4%), followed by Pdyn (28.1%) and Adcyap1 (11.8%). (L) Spatial distribution of PVN projection neurons across clusters. Left: three‐dimensional scatter plot of neuronal soma positions within the PVN (DV–AP–ML coordinate axes), color‐coded by cluster identity. Right: two‐dimensional scatter plot of anterior–posterior (AP) vs. mediolateral (ML) coordinates for each neuron within the PVN. Cluster 1, 3, and 4 neurons are preferentially concentrated in the medial and posterior PVN (AP 600–660 µm range), whereas Cluster 0 is biased toward the anterior subregion and Cluster 2 is broadly distributed along the rostrocaudal axis. This spatial distribution closely corresponds to the mPVN/pPVN subregion identified by TRAP2 imaging as the site of maximal injury‐associated suppression. Data for panels (I–L) were derived from reanalysis of publicly available single‐neuron morphological reconstruction and projection data from Ref. [20]. These panels are reanalyses of 574 PVN neurons with whole‐brain axon reconstruction; no statistical test was applied.

In parallel with behavioral changes, norepinephrine (NE) concentrations at the BTI showed a dynamic elevation following RCI, reaching peak levels at 4wks pi before declining by 8wks pi (Figure 2C). The temporal trajectory of local NE accumulation closely mirrored the severity of anxiety‐like behavior across time points, suggesting that subacute‐phase peripheral sympathetic hyperactivity and central affective disturbance are temporally coupled responses to RCI rather than independent sequelae [9].

To interrogate injury‐associated changes in hypothalamic neural activity at cellular resolution, activity‐dependent labeling was performed using TRAP2 mice combined with whole‐brain optical clearing and light‐sheet fluorescence microscopy. Quantitative mapping revealed altered Fos^TRAP^ density in multiple hypothalamic regions, including the lateral mammillary nucleus (LM, *p* = 0.0129), paraventricular hypothalamic nucleus (PVN, *p* = 0.0281), tuberal nucleus (TU, *p* = 0.0294), lateral hypothalamic area (LHA, *p* = 0.0349), and anterior hypothalamic nucleus (AHN, *p* = 0.0428). Consistent with the subnuclei‐level signatures identified in clinical neuroimaging, among the seven a priori functional subnuclei corresponding to the human hypothalamic atlas used in Figure 1F, the PVN showed the most statistically significant reduction in Fos^TRAP^‐labeled neuron density (*p* < 0.05), with MPO and SOSC reaching trend‐level differences (*p* < 0.1), while the remaining subnuclei showed no significant changes (Figure 2D and Table S4). Three‐dimensional reconstruction of PVN‐localized Fos^TRAP^ signals further demonstrated that the reduction in Fos^TRAP^‐labeled neurons was anatomically concentrated within the medial and posterior PVN subregions (mPVN/pPVN), whereas the anterior subregion (aPVN) was comparatively spared (Figure 2E).

We next recorded in vivo local field potentials (LFP) directly within the PVN across the same post‐injury time course (n = 6, for each group). Broadband (1–100 Hz) PVN LFP power was significantly reduced at 4wks pi relative to uninjured controls (−31.1%; Welch's t = 11.48, P_raw = 8.21 × 10^−^^7^, FDR q = 7.28 × 10^−^^6^), with concordant reductions across the canonical sub‐bands: delta, theta, alpha, beta and high gamma survived FDR correction (q ≤ 0.0409), whereas low gamma showed the same directional reduction without reaching the FDR threshold (q = 0.0569) (Figure 2F–H). Across the full time course, power declined progressively from 2wks pi, reached its nadir at 4wks pi, and partially recovered by 8wks pi (*p* = 0.00195), closely paralleling both the Fos^TRAP^ density findings (Figure 2D) and the behavioral and neurochemical time courses (Figure 2A–C and Figure S1).

Given that PVN neurons are functionally and anatomically heterogeneous, we next sought to determine which projection subtypes within the mPVN/pPVN might be positioned to simultaneously regulate emotional behavior and peripheral autonomic tone. To address this, we performed a reanalysis of publicly available single‐neuron morphological and projection data [20]. K‐medoids clustering based on whole‐brain projection patterns identified five distinct neuronal subpopulations within the PVN (Figure 2I and Figure S2): Cluster 0 (n = 98) and Cluster 1 (n = 200) predominantly innervated local hypothalamic subnuclei, representing short‐range intra‐hypothalamic circuits; Cluster 2 (n = 42) formed medium‐range projections to regions including the periaqueductal gray (PAG) and associated structures implicated in nociception and defensive responses; Cluster 3 (n = 131) and Cluster 4 (n = 103) were characterized by extensive long‐range projections, with 67.2% and 63.1% of their neurons, respectively, extending axons to the spinal cord (Figure 2J,K). Molecular characterization of the 153 identified spinal‐projecting neurons revealed that 46.4% expressed *Oxt*, establishing oxytocinergic neurons as the predominant genotype among PVN neurons capable of engaging spinal autonomic circuitry (Figure 2K).

Critically, spatial mapping of each cluster's somatic distribution within the PVN demonstrated that Cluster 1, 3, and 4 neurons were preferentially concentrated within the medial and posterior PVN (Figure 2L), whereas Cluster 0 was anteriorly biased and Cluster 2 was distributed across the rostrocaudal extent. This anatomical distribution pattern closely corresponds to the injury‐associated reduction in activated neurons observed via TRAP2 imaging, which was itself concentrated in the mPVN/pPVN. Taken together, these results indicate that the neuronal population most affected by RCI—localized to the medial and posterior PVN—largely overlaps anatomically with neurons of Cluster 1, 3, and 4, which collectively possess the anatomical substrate to regulate both descending autonomic output via spinal projections and local intra‐hypothalamic emotional circuitry. Strikingly, *Oxt* represents the predominant molecular marker of these clusters, particularly among spinal‐projecting neurons (46.4% of SC_Proj), providing a cellular‐level convergence with the oxytocin signaling pathway identified as the most biologically relevant enriched pathway in the imaging transcriptomic analysis of RCI patients. The anatomical, functional, and molecular lines of evidence thus converge on PVN^OXT^ neurons as a candidate substrate mechanistically linking emotional disturbance to impaired BTI healing in RCI, motivating their direct experimental interrogation in subsequent analyses.

### 2.3 Retrograde Tracing and Spatial Transcriptomics Identify a PVN Oxytocinergic‐Sympathetic Pathway Regulating BTI Area of Rotator Cuff

To define the central anatomical substrates potentially linked to the rotator cuff, retrograde trans‐synaptic tracing was performed by injecting PRV‐CAG‐EGFP or PRV‐CAG‐mRFP (BrainVTA) into the subchondral region of the supraspinatus enthesis (Figure 3A). Robust retrogradely labeled neurons were detected in the PVN. Quantitative mapping revealed a non‐uniform distribution of PRV‐labeled neurons within the PVN, with significantly higher labeling densities in the medial (mPVN) and posterior (pPVN) subregions compared with the anterior PVN (aPVN) (Figure 3C,D). As an anatomical specificity control, PRV‐labeled neurons were quantified in the supraoptic nucleus (SON) within the same sections. Essentially no retrogradely labeled neurons were detected in either the left or right SON, in clear contrast to the robust labeling in mPVN and pPVN, indicating that retrograde labeling from the enthesis is not a generic property of oxytocin‐rich hypothalamic nuclei but reflects a projection specifically originating in the PVN (Figure 3B).

**FIGURE 3:**
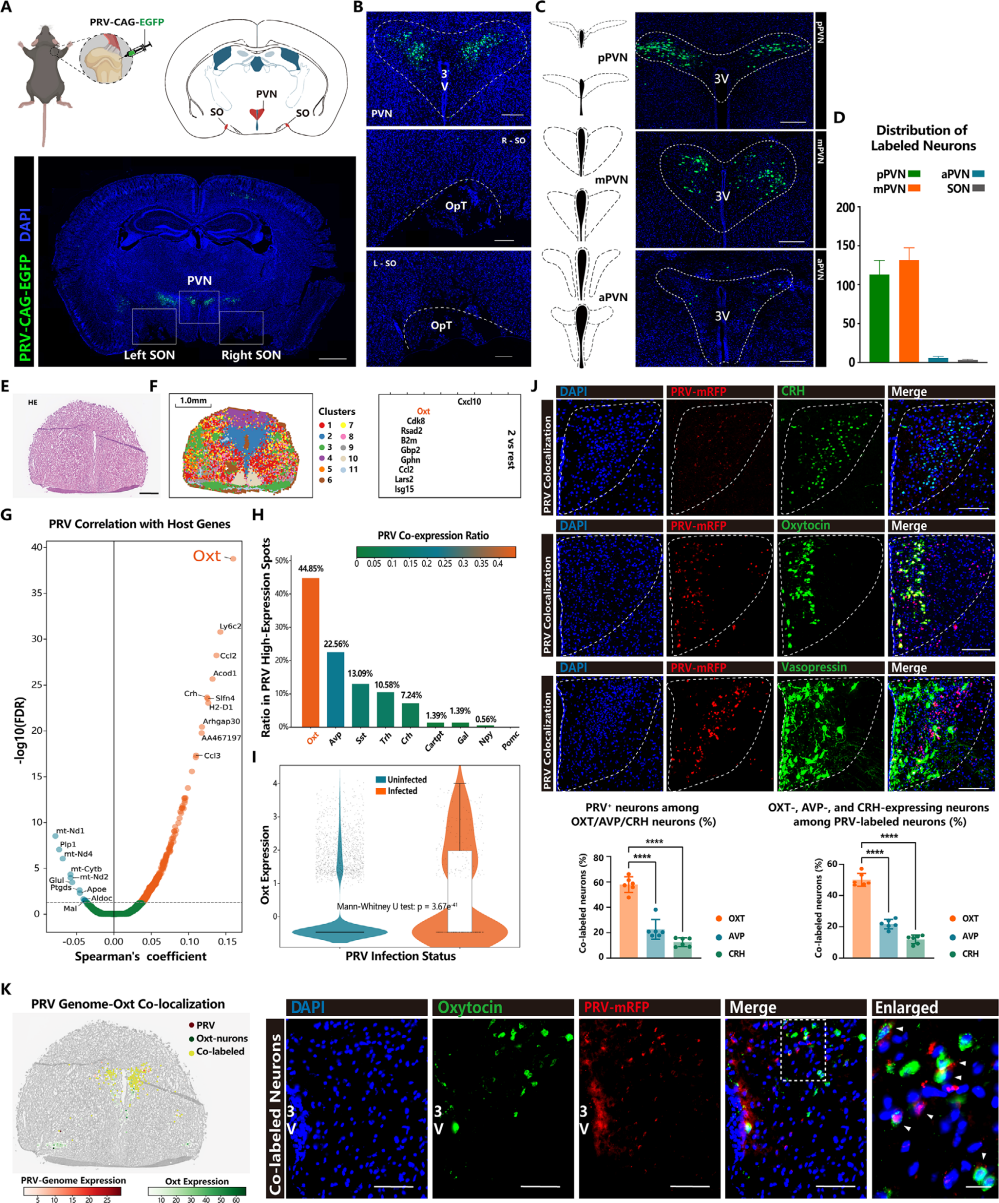
Retrograde viral tracing combined with spatial transcriptomics identifies oxytocinergic neurons in the PVN as a principal central population innervating the rotator cuff enthesis. (A) Schematic of retrograde trans‐synaptic tracing. Upper: pseudorabies virus (PRV‐CAG‐EGFP) was injected into the subchondral region of the supraspinatus enthesis; the coronal schematic indicates the anatomical location of the PVN and the bilateral supraoptic nuclei (SO). Lower: representative low‐magnification coronal section showing PRV‐CAG‐EGFP labeling (green) with DAPI counterstain (blue); boxed regions indicate the PVN and the left and right SON shown at higher magnification in (B). Scale bar = 1 mm. Representative images from n = 6 mice; the same applies to (B), (C) and (K). (B) Higher‐magnification images of the PVN (upper) and of the right (middle) and left (lower) supraoptic nucleus from the same sections, shown as an anatomical specificity control; essentially no PRV‐labeled neurons were detected in either SON. OpT, optic tract; 3 V, third ventricle. Scale bar = 200 µm. (C) Subregion‐specific distribution of PRV‐labeled neurons within the PVN. Left: schematic illustrations of the posterior (pPVN), medial (mPVN), and anterior (aPVN) PVN subregions along the rostrocaudal axis. Right: representative fluorescence images of PRV‐CAG‐EGFP‐labeled neurons at each subregional level, counterstained with DAPI. Scale bar = 200 µm. (D) Quantification of labeled neuron counts per PVN subregion and in the SON (mean ± SEM; n = 6 mice). pPVN and mPVN exhibited significantly higher labeling densities than aPVN, whereas SON labeling was negligible. One‐way ANOVA test. (E) Hematoxylin and eosin (H&E) staining of the coronal section used for spatial transcriptomic profiling at the mPVN level. Scale bar = 1 mm. (F) Left: unsupervised Leiden clustering of the same section identifying 11 spatial clusters; Cluster 2 (orange) recapitulates the anatomical boundaries of the mPVN. Right: top differentially expressed genes defining Cluster 2 (Cluster 2 vs rest), with Oxt as the leading marker alongside antiviral and interferon‐stimulated genes including Cxcl10 and Rsad2. (G) Volcano plot of Spearman correlations between total PRV transcript abundance and host gene expression across spatial spots (x‐axis, Spearman's coefficient; *y*‐axis, −log_10_(FDR)). Oxt (orange) is the most strongly and significantly associated host gene. (H) Proportion of spots expressing each major neuropeptide marker among PRV high‐transcript spots; Oxt accounts for the largest fraction (44.85%), followed by Avp (22.56%) and Sst (13.09%). The color scale indicates the PRV co‐expression ratio. (I) Violin plot comparing Oxt expression between PRV‐infected and uninfected spatial spots, with PRV‐infected spots defined as those exceeding the 95th percentile of the sample‐wide PRV transcript distribution (two‐sided Mann–Whitney U test, p = 3.67 × 10^−^^4^ ^1^). (J) Bidirectional quantification of PRV colocalization with neuropeptide markers. Upper: representative immunofluorescence images of the PVN showing PRV‐CAG‐EGFP or PRV‐CAG‐mRFP together with CRH (top row), oxytocin (middle row), or vasopressin (bottom row) immunoreactivity, counterstained with DAPI. Scale bar = 100 µm. Lower left: proportion of OXT‐, AVP‐, and CRH‐immunoreactive PVN neurons that were PRV‐positive. Lower right: proportion of PRV‐labeled PVN neurons expressing each marker. n = 6 animals per marker, ≥ 3 sections per animal. Data are mean ± SD with individual animals overlaid; one‐way ANOVA with Tukey's post‐hoc test. ^****^ *p* < 0.0001. Raw per‐animal cell counts are provided in Table S8. (K) Spatial and cellular co‐localization of PRV transcripts and oxytocinergic neurons within the PVN. Left: spatial transcriptomic visualization showing the distribution of PRV genome expression (red), Oxt‐expressing spots (green), and co‐labeled spots (yellow) across the mPVN section. Right: representative immunofluorescence images showing co‐localization of PRV‐CAG‐mRFP (red) and oxytocin immunoreactivity (green), counterstained with DAPI (blue); enlarged insets (rightmost panel) highlight individual co‐labeled neurons (arrowheads). Scale bar = 100 µm; enlarged inset scale bar = 20 µm.

To further characterize the cellular identity of injury‐associated PVN neurons, spatial transcriptomic profiling was performed on coronal sections at the mPVN level (Figure 3E). A distinct spatial cluster (cluster 2) was identified, whose distribution closely recapitulated the anatomical boundaries of the mPVN (Figure 3F). This cluster exhibited high expression of *Oxt*, together with enrichment of antiviral and interferon‐related genes, including *Cxcl10* and *Rsad2*. Correlation analysis across spatial spots identified *Oxt* as the host gene most strongly associated with PRV transcript abundance (Figure 3G and Table S6). Consistently, *Oxt* expression was significantly elevated in PRV high–transcript‐abundance spots compared with PRV low–abundance spots (Figure 3I; Mann–Whitney U test, *p* < 0.001). Pattern recognition–based co‐expression analysis further identified a distinct module (pattern 6) characterized by elevated PRV transcript levels and high *Oxt* expression (Figure S3). To determine the neurochemical composition of this retrogradely labeled population quantitatively, we assessed colocalization bidirectionally in n = 6 animals per marker (Figure 3J). Of marker‐positive PVN neurons, 57.9% ± 6.1% of OXT‐immunoreactive neurons were PRV‐positive, significantly exceeding the corresponding proportions for AVP (22.7% ± 7.7%) and CRH (12.5% ± 3.4%) neurons (one‐way ANOVA, F = 93.5, p = 3.4 × 10^−9^). Reciprocally, of all PRV‐labeled PVN neurons, 50.1% ± 4.1% were OXT‐positive, 21.7% ± 3.0% were AVP‐positive, and 11.9% ± 2.8% were CRH‐positive (F = 213.4, p = 9.6 × 10^−12^). These protein‐level proportions closely match the independent spatial‐transcriptomic estimate in Figure 3H, in which Oxt was likewise the most frequent neuropeptide marker among PRV high‐transcript spots (44.85%), followed by Avp (22.56%) (Table S7). Finally, combined spatial transcriptomic visualization and immunofluorescence analysis confirmed the co‐localization of PRV signals with oxytocin‐expressing neurons within the PVN (Figure 3K). Oxytocinergic neurons therefore constitute the largest single identified phenotype among PVN neurons retrogradely connected to the enthesis.

In sum, these results define the temporal window of maximal behavioral alteration, localize injury‐associated central inputs to specific PVN subregions, and identify oxytocinergic neurons in the mPVN as a principal neuronal population linked to the injured rotator cuff.

### 2.4 PVN^OXT^ Neurons Regulate Anxiety‐Like Behavior and Govern BTI Healing Quality in Mice Post‐RCI

To determine whether PVN^OXT^ neurons regulate anxiety‐like behavior and BTI healing after RCI, we selectively manipulated their activity using chemogenetic approaches. AAV‐mediated expression of Gq‐ or Gi‐DREADDs was restricted to OXT‐positive neurons within the PVN. Transduction specificity, quantified across ≥ 3 sections per animal in n = 6 animals per construct, was 89.68% ± 3.97% for Gq‐DREADDs and 90.82 ± 4.24% for Gi‐DREADDs (mean ± SD), with no significant difference between constructs (*p* = 0.64) and the lowest individual animal at 82.95% (Figure 4A,B).

**FIGURE 4:**
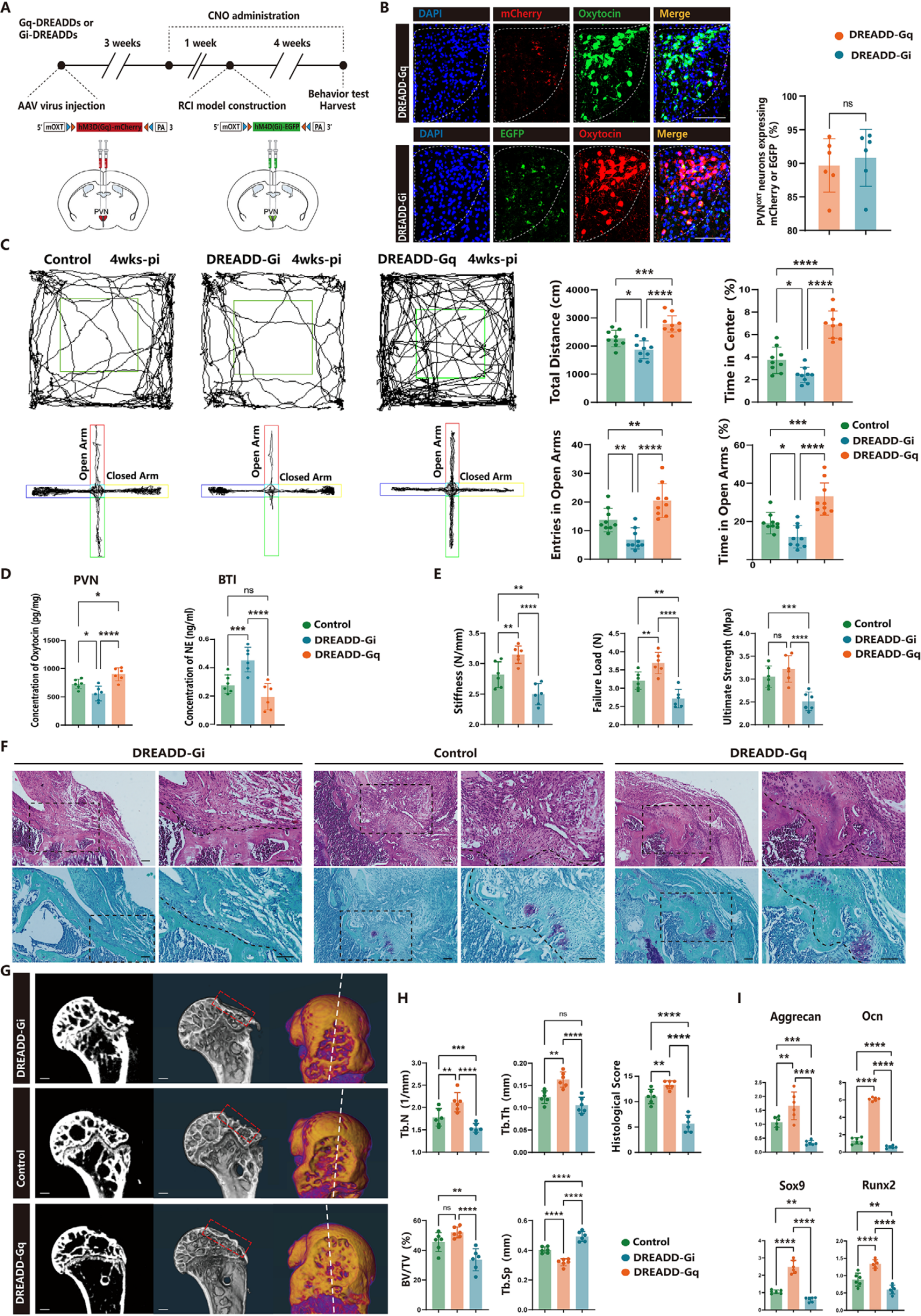
Chemogenetic manipulation of PVN^OXT^ neurons bidirectionally regulates anxiety‐like behavior, site‐specific sympathetic output, and tendon–bone healing quality in RCI mice. (A) Timeline of the experimental procedure. AAV virus encoding Gq‐ or Gi‐DREADDs was injected bilaterally into the PVN four weeks prior to RCI model construction. CNO was administered one week before injury, and behavioral assessments and tissue harvest were performed four weeks post‐injury. The schematic illustrates the viral vector constructs and bilateral injection approach targeting PVN^OXT^ neurons. Control animals received rAAV2/9‐mOXTpr‐EGFP lacking a DREADD coding sequence. (B) Validation of DREADDs expression specificity in PVN^OXT^ neurons. Representative immunofluorescence images show co‐localization of mCherry (Gq‐DREADDs, upper row) or EGFP (Gi‐DREADDs, lower row) with OXT‐immunoreactive neurons in the PVN. The bar graph shows the percentage of total PVN^OXT^ neurons expressing mCherry or EGFP (Gq 89.68% ± 3.97%, Gi 90.82% ± 4.24%; mean ± SD, n = 6 animals per construct, ≥ 3 sections per animal); no significant difference in transduction efficiency was observed between constructs (Welch's *t*‐test, *p* = 0.64). No animal was excluded on the basis of transduction specificity or injection‐site placement. Scale bar = 100 µm. (C) Behavioral assessments using OFT and EPM at 4wks pi. Left: representative locomotor tracking paths (OFT, upper) and arm‐occupancy plots (EPM, lower) for Control, DREADD‐Gi, and DREADD‐Gq groups. Right: quantitative analyses including total distance traveled and time spent in center zone (OFT), and entries into and time spent in open arms (EPM). DREADD‐Gi significantly worsened all behavioral parameters compared with Control; DREADD‐Gq normalized or in some measures exceeded control‐level performance. ^*^ *p* < 0.05, ^**^ *p* < 0.01, ^***^ *p* < 0.001, ^****^ *p* < 0.0001. n = 6 mice per group; data are mean ± SEM with individual animals overlaid; one‐way ANOVA test. Unless otherwise indicated, the same n, data presentation, and test apply to (D)–(I).(D) Neuroendocrine measurements in peripheral and central compartments. Oxytocin concentration was quantified in PVN tissue; NE concentration was quantified at the BTI. Each compartment is presented as an individual bar graph. DREADD‐Gi significantly reduced PVN oxytocin and elevated BTI NE, whereas DREADD‐Gq increased PVN oxytocin; BTI NE in DREADD‐Gq animals was significantly lower than in DREADD‐Gi animals but did not differ from Control (ns). ^*^ *p* < 0.05, ^**^ *p* < 0.01, ^***^ *p* < 0.001, ^****^ *p* < 0.0001. (E) Biomechanical testing of the rotator cuff tendon‐bone complex. Stiffness (N/mm), failure load (N), and ultimate tensile strength (MPa) were compared across Control, DREADD‐Gi, and DREADD‐Gq groups. DREADD‐Gi significantly reduced all parameters; DREADD‐Gq preserved or enhanced biomechanical integrity. ^*^ *p* < 0.05, ^**^ *p* < 0.01, ^***^ *p* < 0.001, ^****^ *p* < 0.0001. (F) Histological evaluation of the BTI. Each left panel shows H&E staining (top) and toluidine blue staining (bottom) of the BTI at 4wks pi across the three groups (DREADD‐Gi, Control, DREADD‐Gq). Each right panel shows magnified views of the boxed regions, with dashed lines marking the injury site. DREADD‐Gi group exhibits disorganized fibrocartilage and reduced proteoglycan deposition; the DREADD‐Gq group shows improved cellular alignment and cartilage formation. Scale bar = 50 µm. Representative images from n = 6 mice per group. (G) Representative micro‐CT images and three‐dimensional reconstructions of the greater tuberosity region of the humerus at 4wks pi across three groups. Scale bar = 1 mm. (H) Quantitative micro‐CT and histological analyses across groups. Including trabecular number (Tb.N, 1/mm), trabecular thickness (Tb.Th, mm), histological score, bone volume fraction (BV/TV, %) and trabecular separation (Tb.Sp, mm). DREADD‐Gi led to significant deterioration in all parameters compared with Control; DREADD‐Gq significantly attenuated these changes. ^*^ *p* < 0.05, ^**^ *p* < 0.01, ^***^ *p* < 0.001, ^****^ *p* < 0.0001, ns = not significant. (I) qRT‐PCR analysis of fibrocartilage‐ and osteogenesis‐related markers (*Aggrecan*, *Ocn*, *Sox9*, *Runx2*) in BTI tissue across groups. DREADD‐Gi significantly suppressed expression of all four markers; DREADD‐Gq preserved their expression at levels exceeding Control. Data are presented as mean ± SEM. ^*^ *p* < 0.05, ^**^ *p* < 0.01, ^***^ *p* < 0.001, ^****^ *p* < 0.0001.

Behavioral analyses revealed that chemogenetic inhibition (DREADD‐Gi) of PVN^OXT^ neurons induced robust anxiety‐like phenotypes, manifested by reduced center exploration and overall locomotor activity in the OFT and decreased open‐arm entries and dwell time in the EPM, whereas chemogenetic activation (DREADD‐Gq) partially reversed these effects (all *p* < 0.05) (Figure 4C).

As an on‐target check that chemogenetic manipulation moved oxytocinergic tone in the intended direction, PVN oxytocin concentration was reduced by DREADD‐Gi and increased by DREADD‐Gq (Figure 4D). In parallel, local NE concentration at the BTI was significantly elevated by DREADD‐Gi, whereas NE levels in DREADD‐Gq animals did not differ significantly from controls (Figure 4D). Circulating serum NE was unchanged by either manipulation, and serum oxytocin was elevated by DREADD‐Gq (Figure S4), indicating site‐specific autonomic control of the BTI microenvironment rather than a generalized shift in systemic sympathetic tone.

Functionally, DREADD‐Gi significantly impaired BTI healing quality, as indicated by reduced stiffness, failure load, and ultimate tensile strength of the tendon‐bone complex, whereas DREADD‐Gq preserved or enhanced these biomechanical parameters (Figure 4E). Histological analyses further demonstrated that PVN^OXT^ neuron suppression disrupted fibrocartilaginous regeneration at the BTI, characterized by disorganized cellular alignment, reduced cartilage formation, and aberrant fibrous tissue architecture resulting in marked interface degeneration (Figure 4F). In parallel, micro‐CT analyses revealed that PVN^OXT^ neuron suppression led to pronounced deterioration of bone microarchitecture at the greater tuberosity, including decreased trabecular number, thickness, and bone volume fraction with increased trabecular separation, whereas DREADD‐Gq significantly attenuated these changes (Figure 4G,H). At the molecular level, expression of fibrocartilage‐ and osteogenesis‐related markers (*Aggrecan*, *Sox9*, *Ocn*, and *Runx2*) was significantly suppressed following DREADD‐Gi, whereas DREADD‐Gq preserved their expression (Figure 4I).

Collectively, these results indicate that PVN^OXT^ neuron activity bidirectionally influences both emotional behavioral state and the structural and biomechanical quality of BTI healing after RCI, in association with site‐specific modulation of local sympathetic neurotransmitter levels at the tendon–bone interface.

### 2.5 Sympathetic Relay Links PVN^OXT^ Neurons to Peripheral Autonomic Output

To delineate the efferent pathway responsible for mediating the effects of PVN^OXT^ neurons on BTI healing, we next examined the anatomical and functional organization of the PVN–IML–SCG circuit. Retrograde viral tracing from the enthesis robustly labeled sympathetic chain components, including the superior cervical ganglion (SCG), the stellate ganglion (SG), and T2 sympathetic ganglia, as well as sympathetic preganglionic neurons within the IML, confirming the integrity of a spinal sympathetic relay downstream of hypothalamic inputs. Co‐localization of PRV signals with tyrosine hydroxylase (TH) immunoreactivity in the SCG further verified the sympathetic identity of retrogradely labeled neurons (Figure 5A).

**FIGURE 5:**
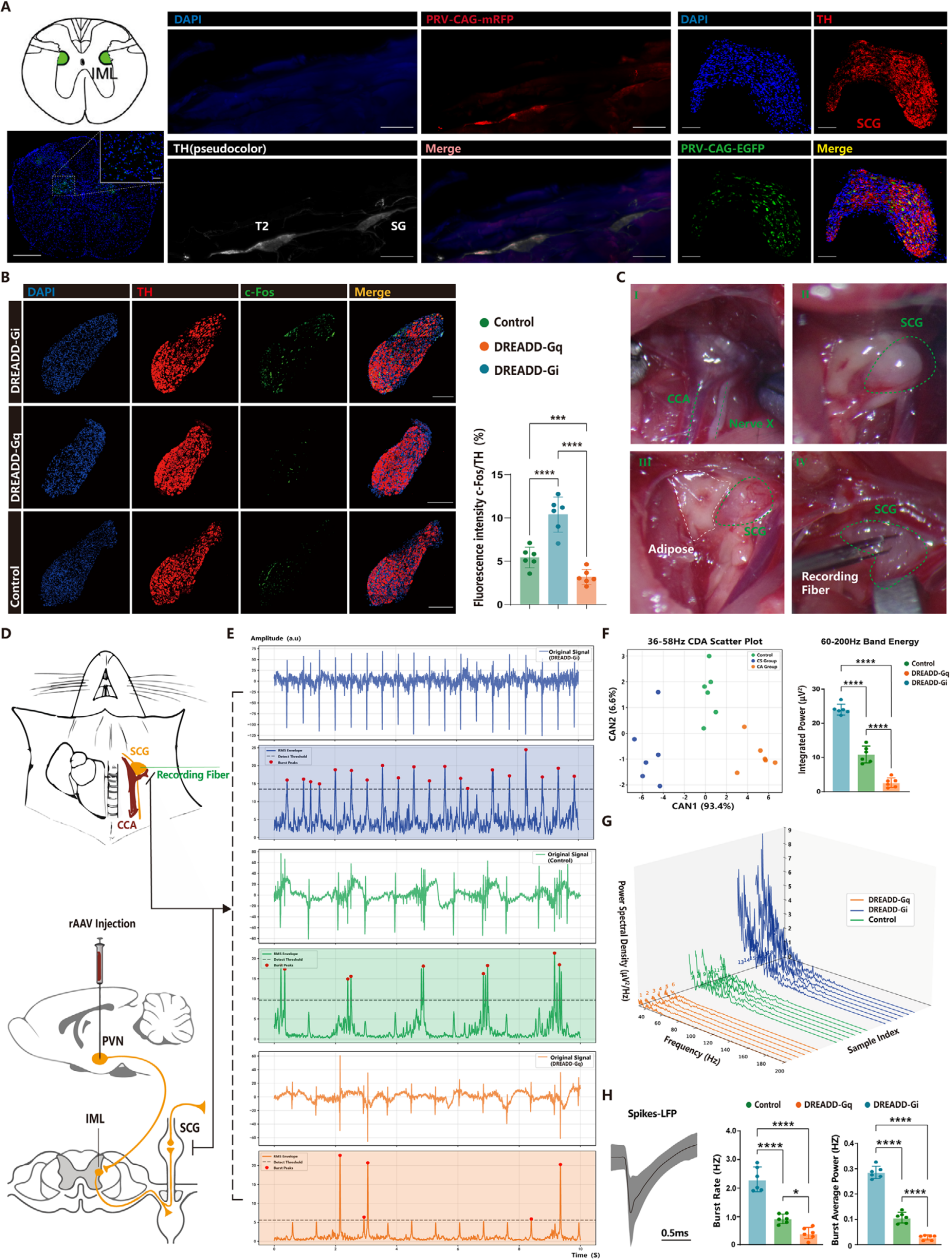
Identification and functional validation of the PVN–IML–SCG sympathetic circuit. (A) Retrograde labeling of the PVN‐IML‐SCG sympathetic circuit. Left: schematic location of the spinal cord IML and a representative section showing PRV‐labeled neurons in the IML; the boxed region is shown at higher magnification in the inset. Middle: representative fluorescence images showing PRV‐CAG‐mRFP‐labeled neurons in the stellate ganglion (SG) and T2 ganglia indicated (scale bar = 500 µm). Right: representative fluorescence images of the SCG showing co‐localization of PRV‐CAG‐EGFP with tyrosine hydroxylase (TH) immunoreactivity, confirming the sympathetic identity of retrogradely labeled SCG neurons (scale bar = 100 µm). (B) Immunofluorescence analysis of SCG neuronal activation across experimental groups. Representative images of TH (red), c‐Fos (green), DAPI (blue), and merged channels are shown for the DREADD‐Gi, DREADD‐Gq, and Control groups (top to bottom). Bar graph: fluorescence intensity ratio of c‐Fos to TH (%) across groups; DREADD‐Gi showed the highest activation, DREADD‐Gq the lowest. Scale bar = 200 µm. ^*^ *p* < 0.05, ^**^ *p* < 0.01, ^***^ *p* < 0.001, ^****^ *p* < 0.0001. n = 6 mice per group; data are mean ± SEM; one‐way ANOVA test. Unless otherwise indicated, the same n, data presentation, and test apply to (E)–(H); images in (A) and (C) are representative of the same animals. (C) Surgical implantation of SCG recording electrodes. (i) Exposure of the left common carotid artery (CCA) with the vagus nerve (CN X) visible. (ii) Identification and full exposure of the SCG posterior to the carotid bifurcation. (iii) Microdissection of the SCG from surrounding adipose tissue and fascial attachments. (iv) Precision implantation of a tungsten microelectrode into the SCG parenchyma. (D) Schematic overview of the experimental paradigm. rAAV was injected into the PVN, and sympathetic neural activity was recorded from the SCG via an implanted tungsten microelectrode. (E) Representative local field potential (LFP) recordings from the SCG in the DREADD‐Gi (blue), Control (green), and DREADD‐Gq (orange) groups. Raw signals (top of each trace) and corresponding spike‐detected signals (bottom of each trace, red dots indicating detected spikes) are shown. (F) Left: Canonical Discriminant analysis (CDA) scatter plot showing frequency bands with significant group differences; prominent separation among groups is observed within the 36–58 Hz range. Right: quantification of integrated power in the 60–200 Hz frequency band across groups; DREADD‐Gi is significantly elevated and DREADD‐Gq is significantly reduced relative to Control. *p* < 0.0001. (G) Three‐dimensional power spectral density (PSD) plots of SCG LFPs across the 36–200 Hz frequency range, illustrating distinct spectral signatures among control, DREADD‐Gq, and DREADD‐Gi group. (H) Spike‐sorting analysis of SCG neural activity. Left: Representative spike‐sorted waveforms (scale bar = 0.5 ms). Middle: burst rate (Hz) across groups. Right: burst average power (Hz) across groups. DREADD‐Gi significantly increased both parameters; DREADD‐Gq markedly diminished both. Data are presented as mean ± SEM. ^*^ *p* < 0.05, ^**^ *p* < 0.01, ^***^ *p* < 0.001, ^****^ *p* < 0.0001.

Among the labeled ganglia, the SCG was the most robustly and consistently labeled and was therefore selected for functional interrogation (see Section 4). We assessed whether chemogenetic manipulation of PVN^OXT^ neurons altered SCG neuronal activity. Immunofluorescence analyses revealed that DREADD‐Gi significantly increased c‐Fos expression in TH‐positive SCG neurons at 4wks pi, whereas DREADD‐Gq markedly reduced SCG neuronal activation compared with control animals (Figure 5B), indicating bidirectional, state‐dependent regulation of sympathetic outflow by PVN^OXT^ neurons.

To directly interrogate sympathetic neural dynamics, in vivo electrophysiological recordings were performed by implanting microelectrodes into the SCG (Figure 5C,D). Representative local field potential (LFP) recordings demonstrated that DREADD‐Gi markedly enhanced SCG neural activity, characterized by increased spike density and bursting events, whereas DREADD‐Gq produced a pronounced suppression of sympathetic firing at 4wks pi (Figure 5E). Canonical Discriminant Analysis (CDA) was first employed to unbiasedly identify frequency bands exhibiting significant group differences, revealing a prominent separation among groups within the 36–58 Hz range (Figure 5F). Notably, given that high‐frequency LFP components are closely associated with sympathetic multi‐unit activity and bursting dynamics, we further quantified power in the 60–200 Hz frequency band as a functionally relevant index of sympathetic output. Consistent with spike‐level observations, integrated power within this high‐frequency range was significantly elevated in the DREADD‐Gi group and markedly reduced in the DREADD‐Gq group relative to controls (Figure 5F). Power spectral density (PSD) analysis further revealed distinct spectral signatures across groups, with prominent high‐frequency power enrichment following PVN^OXT^ neuron suppression and a generalized attenuation of spectral power after activation (Figure 5G). Spike‐sorting analyses corroborated these findings, demonstrating that DREADD‐Gi significantly increased sympathetic burst rate and burst average power, whereas DREADD‐Gq markedly diminished both parameters at 4wks pi (Figure 5H).

In aggregate, these results identify the PVN–IML–SCG pathway as a functional sympathetic circuit through which PVN^OXT^ neurons exert bidirectional control over peripheral autonomic activity, providing a circuit‐level substrate through which hypothalamic oxytocin signaling engages sympathetic regulation in the context of RCI.

### 2.6 SCG Acts as a Functional Peripheral Relay Mediating PVN^OXT^‐Dependent Regulation of BTI Healing

Building on the anatomical and functional evidence implicating the PVN–IML–SCG sympathetic pathway, we examined the contribution of SCG sympathetic neurons to tendon–bone healing both with and without PVN DREADD‐Gi inhibition. Four groups of mice underwent rotator cuff injury: Control, DREADD‐Gi, DREADD‐Gi+SCG_Abl, and SCG_Abl (n = 6 mice per group for each quantitative outcome in Figure 6C–F). Local diphtheria toxin administration markedly reduced tdTomato‐positive SCG neurons compared with the non‐ablated control condition (Figure 6A,B).

**FIGURE 6:**
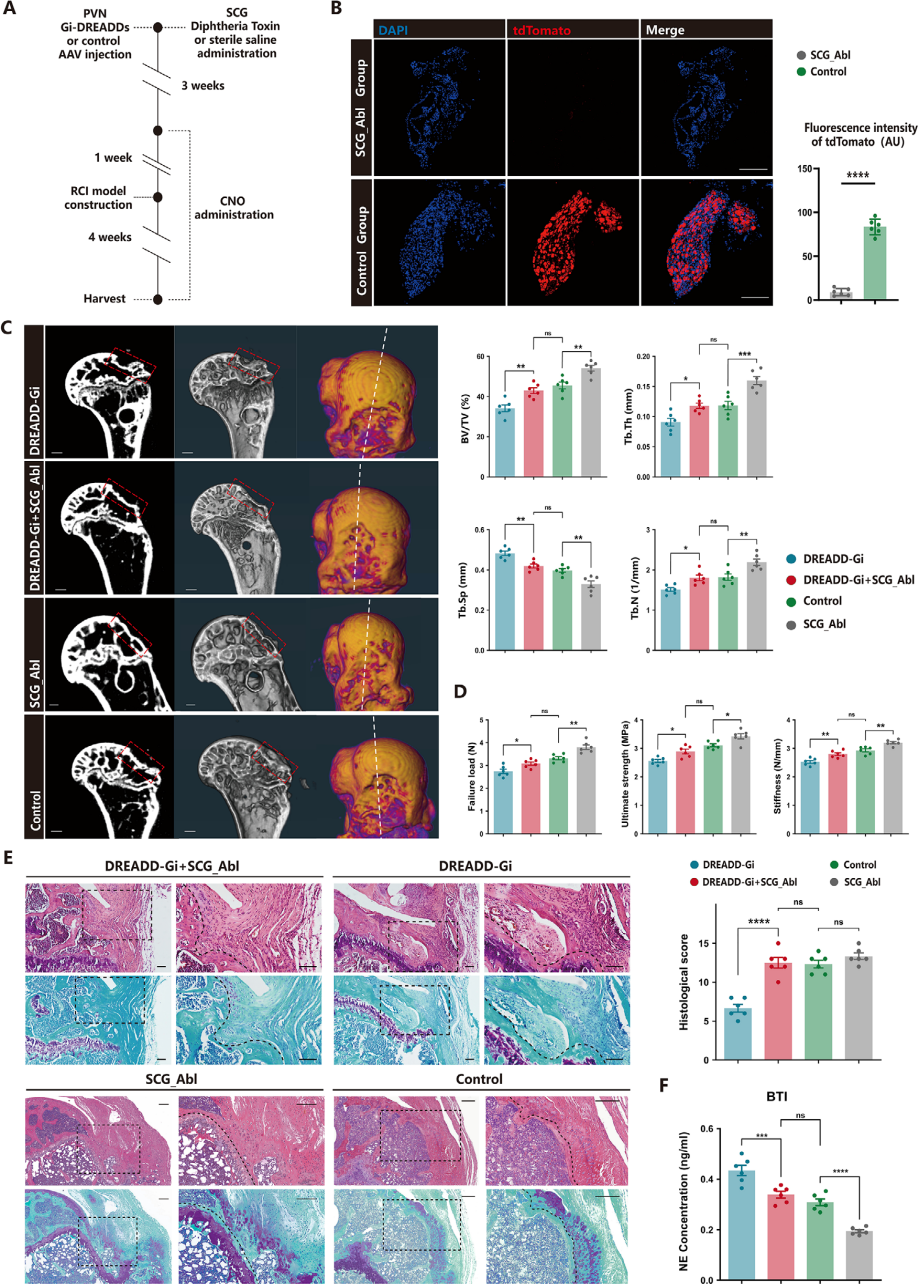
SCG sympathetic neuron ablation attenuates DREADD‐Gi‐associated impairment of tendon–bone healing. (A) Experimental timeline and four‐group design. Th‐Cre;Rosa26‐LSL‐iDTR‐tdTomato mice received either PVN Gi‐DREADD or control AAV and either local SCG diphtheria toxin or saline, generating Control, DREADD‐Gi, DREADD‐Gi+SCG_Abl, and SCG_Abl groups. All groups underwent rotator cuff injury, and tissues were collected four weeks after injury. (B) Validation of SCG sympathetic neuron ablation. Representative SCG sections from SCG_Abl and Control mice show DAPI, tdTomato, and merged fluorescence images. The graph quantifies SCG tdTomato fluorescence intensity. n = 6 mice per group; two‐tailed Welch's *t*‐test. (C) Micro‐CT analysis of greater tuberosity bone microarchitecture four weeks after injury. Representative two‐dimensional sections and three‐dimensional reconstructions are shown for all four groups. Graphs show bone volume fraction (BV/TV), trabecular thickness (Tb.Th), trabecular separation (Tb.Sp), and trabecular number (Tb.N). Scale bar = 1 mm. (D) Biomechanical testing of the supraspinatus tendon–bone complex. Failure load, ultimate tensile strength, and stiffness are compared across the four groups. (E) Histological evaluation of the BTI. Representative H&E and toluidine blue images, including overview and higher‐magnification views, are shown for all four groups. Dashed lines mark the repair interface. The graph shows histological healing scores. Scale bar = 50 µm. (F) Local NE concentration at the BTI across the four groups. For each outcome in (C)–(F), n = 6 mice per group. Data are presented as mean ± SEM with individual animals overlaid. Four‐group comparisons used one‐way ANOVA followed by Tukey's multiple‐comparison test. ^*^ *p* < 0.05, ^**^ *p* < 0.01, ^***^ *p* < 0.001, ^****^ *p* < 0.0001; ns, not significant.

At four weeks after injury, adding SCG ablation to DREADD‐Gi inhibition improved all four micro‐CT indices, the histological healing score, and all three biomechanical measures compared with DREADD‐Gi inhibition alone (all Tukey‐adjusted *p* < 0.05; Figure 6C–E). Local BTI NE concentration was also lower in the DREADD‐Gi+SCG_Abl group than in the DREADD‐Gi group (adjusted *p* = 0.000576; Figure 6F).

SCG ablation alone, without PVN DREADD‐Gi inhibition, improved all four micro‐CT indices and all three biomechanical measures relative to injured controls (all adjusted *p* < 0.05; Figure 6C,D). The histological score did not differ significantly between SCG_Abl and Control mice (adjusted *p* = 0.550; Figure 6E), whereas BTI NE concentration was lower after SCG ablation (adjusted *p* = 0.000072; Figure 6F). Together, these findings show that SCG sympathetic neurons contribute to BTI healing both independently and in the setting of PVN oxytocinergic neuron inhibition.

## 3 Discussion

### 3.1 Anxiety Emerges as the Predominant Psychological Phenotype in RCI and May be Functionally Linked to Hypothalamic Suppression

RCI is the most common cause of shoulder pain and disability, and is increasingly recognized as a condition accompanied by substantial psychological burden. The clinical relevance of this burden is amplified by the observation that preoperative psychological comorbidities are well‐established independent predictors of postoperative pain, function, and quality‐of‐life outcomes after rotator cuff repair [2, 4, 21, 22, 23, 24]. In the present clinical cohort, anxiety was highly prevalent (27.9%) among patients with RCI, exceeding rates reported in the general population (∼4%) [25] and in other musculoskeletal disorders such as osteoarthritis (21.3%) [26]. Importantly, preoperative anxiety has been repeatedly identified as an independent predictor of inferior postoperative pain, function, and quality‐of‐life outcomes after rotator cuff repair, irrespective of tear severity or surgical technique. This positions anxiety not as an incidental psychological accompaniment of RCI but as a clinically actionable target whose perioperative recognition and management may directly influence surgical outcome.

Despite this prognostic relevance, the central origin of RCI‐associated anxiety has remained unaddressed. Our results suggested that although the canonical pain–affective regions (dACC, amygdala, insula) also exhibited group‐level differences between patients and controls, only the bilateral hypothalamus showed individual‐level coupling to symptom severity, identifying the hypothalamus as the central region most closely tracking affective symptom burden in our cohort. Beyond identifying a focal hypothalamic substrate, the convergence of subnuclei‐resolved functional mapping with spatially corrected imaging‐transcriptomic enrichment of oxytocin signaling provides a molecular entry point for mechanistic interrogation. To our knowledge, this constitutes the first patient‐level demonstration that RCI‐associated anxiety has a definable central neurobiological correlate, converting a long‐standing clinical observation—that anxiety predicts post‐repair outcomes—into a tractable circuit hypothesis. Because the clinical data are cross‐sectional, they cannot establish whether hypothalamic suppression precedes or follows anxiety onset. It remains possible that pain, or injury‐related sleep disturbance and chronic stress, drives both the anxiety and the hypothalamic signal change, rather than hypothalamic dysfunction driving anxiety directly. The directionality implied by our animal experiments should therefore be regarded as a mechanistic hypothesis tested in the mouse, not as an inference established in patients. The convergence between an anatomically focal functional signal and dominant signaling pathways, rather than a diffuse pattern across multiple programs, motivated us to ask whether this hypothalamic–oxytocinergic correlate is mechanistically engaged in tendon–bone repair, an inference that can only be tested in animal models.

These clinical observations were faithfully recapitulated in mice. RCI‐induced anxiety‐like behavior peaked at 4 weeks post‐injury (4wks pi), with concordant changes across non‐redundant OFT and EPM parameters more parsimoniously explained by genuine affective alteration than by locomotor confounds. Critically, this temporal trajectory closely mirrored the dynamic elevation of NE at the BTI, defining a subacute window in which central affective disturbance and peripheral sympathetic activation are co‐regulated and mechanistically tractable.

### 3.2 PVN^OXT^ Neurons Coordinate Emotional Regulation and Tendon–Bone Healing Through a Defined PVN–IML–Sympathetic Chain Axis

Our focus on oxytocinergic signaling was not specified a priori. It emerged empirically from an unbiased, genome‐wide imaging‐transcriptomic screen, in which the oxytocin signaling pathway ranked among the top spatially concordant, FDR‐significant hits when the hypothalamic zALFF differential map was tested against the full KEGG pathway library, and was independently reinforced by reanalysis of single‐neuron projectome data identifying Oxt as the dominant neuropeptide marker among PVN neurons capable of engaging spinal autonomic circuitry. Building upon these observations, we then identified PVN^OXT^ as a critical neural substrate linking affective regulation with peripheral tissue repair. Three independent lines of evidence converged on this population as the integrative substrate of this brain–body coupling. First, TRAP2‐based whole‐brain activity mapping demonstrated that the PVN exhibited the most significant injury‐associated reduction in activated neurons among hypothalamic subnuclei, with the reduction anatomically concentrated in the medial and posterior PVN (mPVN/pPVN)—directly mirroring the subnuclei‐level signatures identified in clinical neuroimaging. Second, reanalysis of recent single‐neuron connectome data [20] revealed that PVN clusters preferentially distributed in the mPVN/pPVN comprise both long‐range spinal‐projecting neurons engaging descending autonomic relays and intra‐hypothalamic projection neurons innervating local emotion‐related subnuclei, with *Oxt* as the dominant neuropeptide identity. This dual projection architecture provides a parsimonious anatomical substrate for the simultaneous regulation of autonomic output and affective state by a single neuronal population. Third, retrograde trans‐synaptic tracing from the supraspinatus enthesis selectively labeled the mPVN/pPVN, and Stereo‐seq spatial transcriptomics identified *Oxt* as the host gene most strongly correlated with PRV transcript abundance, with direct immunofluorescent co‐localization. The activity‐defined, projection‐defined, and tracer‐defined evidence thus converge on the same mPVN/pPVN^OXT^ population, providing converging anatomical and molecular grounds motivating direct causal interrogation in the mouse model.

Chemogenetic manipulation tested this role bidirectionally. Selective inhibition of PVN^OXT^ neurons induced anxiety‐like behavior and impaired BTI healing across structural, histological, molecular, and biomechanical parameters, whereas activation produced opposing effects. Mechanistically, PVN^OXT^ manipulation altered local BTI NE levels without affecting circulating serum NE, indicating a site‐specific autonomic control rather than a generalized shift in systemic sympathetic tone. These single‐time‐point concentration measurements are intended as directional manipulation checks rather than as evidence of mediation; they do not identify which neuronal subpopulation or projection produces the peripheral effect, an attribution that rests instead on the anatomical, circuit‐functional, and loss‐of‐function evidence presented in Figures 3, 5, and 6.

The downstream substrate was identified as the PVN–IML–sympathetic chain pathway, with retrograde labeling of the IML, SCG, stellate, and T2 ganglia and TH co‐localization confirming sympathetic identity.

Two complementary readouts validated bidirectional pathway control by PVN^OXT^ neurons: ex vivo c‐Fos in TH^+^ SCG neurons paralleled PVN^OXT^ activity state, while in vivo SCG electrophysiology recapitulated this pattern at finer temporal resolution—suppression elevated spike density, bursting rate, and LFP power, with activation producing the inverse. Selective DT‐mediated ablation of TH‐positive SCG neurons improved the structural, histological, and biomechanical deficits associated with PVN DREADD‐Gi inhibition and reduced local BTI NE. SCG ablation alone also improved bone microarchitecture and biomechanical performance and lowered BTI NE in injured mice. These complementary comparisons establish a functional contribution of SCG sympathetic neurons to BTI repair both with and without PVN oxytocinergic neuron inhibition. This loss‐of‐function step identifies the SCG as a peripheral relay necessary for the transduction of central oxytocinergic dysregulation into impaired tendon–bone repair, while leaving open a parallel contribution from the stellate and T2 ganglia, which were also retrogradely labeled. A cervical sympathetic contribution to shoulder innervation is further supported by classical anatomical tracing of the sympathetic and sensory supply of the shoulder joint [27], which motivated our focus on the SCG.

The inhibitory direction of PVN^OXT–^sympathetic coupling observed here aligns with Pan et al., who showed that PVN^OXT^ neuron stimulation reduces c‐Fos in the celiac–superior mesenteric ganglia and decreases circulating NE [28]; our findings extend this inhibitory coupling to the musculoskeletal compartment with circuit‐level resolution at both ganglionic (SCG) and tissue (BTI) levels. By contrast, Roy et al. reported that PVN^OXT^ activation increases cardiac sympathetic nerve activity following myocardial infarction [29], suggesting a directionally opposite coupling for cardiac sympathetic outflow.

This organ‐specific divergence likely reflects differential preganglionic relays, target‐specific neuropeptide co‐transmission, and the morphological heterogeneity of oxytocin neurons [30]. Oxytocin neurons are broadly divided into magnocellular (MagnOT) and parvocellular (ParvOT) subtypes; ParvOT spinal‐projecting autonomic neurons (SPANs) target the IML and primarily govern autonomic output, whereas MagnOT neurons predominantly exert neuroendocrine functions via posterior pituitary projections with limited spinal collateralization [12, 31, 32, 33]. Consistent with this, chemogenetic activation also elevated circulating oxytocin (Figure S4), indicating concurrent engagement of magnocellular neuroendocrine oxytocin neurons by the mOXT‐promoter‐driven construct. The mOXT promoter used in our DREADD constructs labels both subpopulations, and the precise contribution of ParvOT‐SPAN vs. MagnOT neurons to the BTI‐directed effect remains to be dissected with subtype‐restricted intersectional approaches. In addition, oxytocin has been characterized as an osteoanabolic factor within the hypothalamic–pituitary–bone axis [34, 35, 36], although serum oxytocin was elevated in our chemogenetic activation group, the relative contributions of centrally derived vs. locally produced osteoblast‐derived oxytocin to enthesis remodeling cannot be fully resolved by the present design.

### 3.3 A Brain–Body Framework for RCI, Clinical Implications, and Limitations

Collectively, the clinical and experimental data support an integrated brain–body model in which RCI is accompanied by suppression of PVN^OXT^ neurons; this central oxytocinergic dysfunction is functionally coupled with anxiety‐like affective state and, through disinhibition of the IML–SCG sympathetic relay, drives elevated local NE at the BTI that compromises the structural and biomechanical quality of tendon–bone repair. To our knowledge, this study provides the first evidence that manipulating a defined central neuronal population—PVN^OXT^ neurons—bidirectionally influences both anxiety‐like behavior and musculoskeletal healing after rotator cuff injury. Anxiety in RCI is thus not a secondary psychological burden but a mechanistically coupled component of the repair process, supporting a shift from purely tissue‐focused treatment toward integrated neuromodulatory and tissue‐targeted strategies. Modalities engaging hypothalamic oxytocinergic signaling—pharmacological, behavioral, or non‐invasive neurostimulation—may concurrently alleviate anxiety and improve healing outcomes, and warrant prospective evaluation in perioperative rehabilitation paradigms.

Several limitations should be acknowledged. First, our clinical assessment relied on the HADS. HADS is a validated screening instrument rather than a structured diagnostic interview, and assessment was conducted in a hospital setting; community‐based or pre‐admission assessment, together with a structured diagnostic interview, would help dissociate injury‐specific from hospitalization‐related anxiety. Second, our cross‐sectional clinical neuroimaging cannot establish whether hypothalamic suppression precedes or follows anxiety onset; longitudinal perioperative cohorts would clarify the temporal direction and assess whether hypothalamic activity predicts surgical outcome. Third, all mouse experiments were conducted in adult males; given sex differences in oxytocin signaling and pain processing, generalizability to females requires dedicated investigation. Fourth, behavioral characterization relied on the OFT and EPM. Total locomotor distance was itself reduced at 4wks pi, and although the spatially selective nature of the changes and their temporal coupling to independent central and peripheral measures argue against generalized hypolocomotion as the sole explanation, orthogonal anxiety assays with less dependence on ambulation — such as the light–dark box or novelty‐suppressed feeding — would provide valuable confirmation. Fifth, the mOXT‐driven DREADD strategy does not distinguish ParvOT‐SPAN from MagnOT neurons, and intersectional genetic or projection‐defined approaches (e.g., retrograde Cre combined with OXT‐flp) will be needed to assign subtype‐specific contributions to BTI healing. Relatedly, although chemogenetic activation increased circulating oxytocin, the present design does not separate central PVN‐derived from peripheral osteoblast‐derived oxytocin contributions to enthesis remodeling. Sixth, PRV‐Bartha is a polysynaptic tracer that cannot definitively assign first‐order vs. higher‐order synaptic positions; complementary monosynaptic tracers (e.g., rabies‐ΔG) will refine circuit‐level mapping. Retrograde tracing also labeled the stellate and T2 ganglia in addition to the SCG; the SCG ablation experiment therefore establishes the necessity of this relay rather than its exclusivity. Although SCG ablation lowered NE at the tendon–bone interface and improved several measures of repair, it removes the activity of a sympathetic neuronal population and may also alter cotransmission, vascular regulation, and local immune responses. We therefore cannot conclude that NE is the unique or necessary molecular mediator without a selective local adrenergic intervention or NE add‐back experiment. Oxytocin may also influence BTI repair through its effects on inflammatory signaling. Previous work has linked oxytocin receptor activation to anti‐inflammatory and proimmune transcriptional responses [37]. During tendon–bone healing, OXT‐dependent immune regulation could act alongside SCG‐associated changes in local NE. Although serum oxytocin increased after DREADD‐Gq activation in our study, local inflammatory cell composition, cytokine expression, and OXTR signaling at the BTI were not assessed. The relative contribution of these processes to repair therefore remains to be defined. Also, the TRAP2‐based whole‐brain Fos mapping was performed with a small sample size (n = 3 mice per group) at a single post‐injury time point. Accordingly, comparisons across the 28 surveyed hypothalamic nuclei were treated as exploratory (unadjusted *p*‐values), and the trend‐level differences (*p* < 0.1) in several nuclei (e.g., MPO, SOSC) represent candidate effects that warrant validation in larger cohorts. Finally, our analyses focused on the subacute window (4wks pi); long‐term durability of healing improvements and potential rebound effects after withdrawal of neuromodulatory intervention remain to be determined. Future studies integrating high‐resolution hypothalamic imaging or autonomic readouts (e.g., heart rate variability, microneurography) with rehabilitation outcomes could further strengthen this translational bridge.

A further source of heterogeneity concerns the neurochemical identity of the retrogradely labeled population itself. Although oxytocinergic neurons constituted the largest identified phenotype among PRV‐labeled PVN neurons (50.1%), vasopressinergic (21.7%) and corticotropin‐releasing (11.9%) neurons were also appreciably represented (Figure 3J). Both AVP and CRH neurons are established components of PVN pre‐autonomic circuitry, and a parallel contribution from these populations to sympathetic regulation of the enthesis cannot be excluded by the present design; dissociating their respective contributions will require cell‐type‐specific manipulation of each population. CRH, vasopressinergic and noradrenergic contributions to RCI‐associated anxiety therefore remain plausible and complementary, and are not excluded by the present work.

## 4 Methods

### 4.1 Participants

The clinical study was reviewed and approved by the Medical Ethics Committee of Xiangya Hospital, Central South University (approval no. 202307601), and was conducted in accordance with the Declaration of Helsinki. All participants provided written informed consent.

Inclusion criteria for the RCI group: age 20–75 years; RCI confirmed by X‐ray and MRI; no prior diagnosis of mental illness; ability to complete follow‐up with complete data. Exclusion criteria: pain in regions other than the shoulder; history of shoulder dislocation, fracture, or surgery; metallic implants or claustrophobia precluding MRI; cerebrovascular or central nervous system disorders; cognitive or communication impairment; long‐term use of sedative or hypnotic drugs; any other condition deemed unsuitable by the investigators. Healthy controls were age‐ and sex‐matched volunteers free of shoulder, neurological, or psychiatric disease.

Based on these criteria, 48 patients with RCI and 31 healthy controls were initially enrolled. In the RCI group, 2 participants were excluded due to incomplete clinical or questionnaire data, 1 was excluded after MRI revealed organic brain lesions, and 2 were excluded due to excessive head motion during rs‐fMRI scanning (translation > 2 mm or rotation > 2°). In the control group, 1 participant was excluded for the same head motion criterion. A total of 43 patients with RCI and 30 healthy controls were ultimately included in all clinical analyses. There were no significant differences in age or sex distribution between the final included groups.

### 4.2 Clinical Assessment

General information (age, gender), medical history, physical examination, and shoulder imaging (X‐ray, MRI) were collected. Anxiety and depression were quantified using the Hospital Anxiety and Depression Scale (HADS). HADS‐A and HADS‐D scores were classified as Negative (0–7), Mild (8–10), Moderate (11–14), or Severe (15–21). Group differences in HADS scores were evaluated by the two‐tailed Mann–Whitney U test. HADS‐A and HADS‐D are independent seven‐item subscales validated to load onto distinct anxiety and depression factors and were analyzed separately.

### 4.3 Resting‐State fMRI Acquisition

rs‐fMRI was acquired on a 3.0 T scanner (Discovery MR750w, GE Healthcare) equipped with a phased‐array head coil; foam padding and earplugs were used to minimize scanner noise and head motion.

T1‐weighted structural imaging (BRAVO sequence): TR/TE shortest, FOV 25.6 cm, slice thickness 1 mm with no gap, flip angle 12°, matrix 256 × 256, 190 slices.

EPI‐BOLD functional imaging: TR 2000 ms, TE 28 ms, matrix 60 × 60, FOV 21.0 cm, slice thickness 3.5 mm, inter‐slice gap 0.5 mm, flip angle 90°, 38 slices, 240 volumes.

During scanning, subjects lay supine in a quiet environment with eyes closed, were instructed to remain awake and refrain from systematic thought, and were debriefed after scanning to confirm wakefulness.

### 4.4 fMRI Preprocessing and zALFF Analysis

Data were processed in MATLAB R2018b using SPM12 and DPABI/DPARSFA. Preprocessing comprised: (i) discarding the first 10 volumes; (ii) slice‐timing correction; (iii) head‐motion correction (subjects with translation > 2 mm or rotation > 2° excluded); (iv) coregistration of structural and functional images and segmentation; (v) normalization to MNI space using DARTEL; (vi) spatial smoothing (Gaussian kernel, FWHM 6 mm); (vii) linear detrending; (viii) nuisance regression of Friston‐24 head‐motion parameters and white‐matter/CSF signals; (ix) band‐pass filtering (0.01–0.08 Hz).

The amplitude of low‐frequency fluctuation (ALFF) was computed at each voxel as the square root of integrated power within 0.01–0.08 Hz, then z‐standardized within each subject to yield zALFF maps. Mean zALFF was extracted from the 110 grey‐matter ROIs (atlas label type 1; 55 per hemisphere) of the 189‐region Johns Hopkins University (JHU) brain atlas [17]. Two‐sample *t*‐tests with age and sex as covariates compared zALFF between RCI and control groups, with Benjamini–Hochberg FDR correction (q < 0.05) across the 110 grey‐matter ROIs. Spearman correlations between ROI‐level zALFF and individual HADS‐A scores were computed within RCI patients (n = 43); resulting *p*‐values were FDR‐corrected across the six FDR‐significant group‐difference regions.

### 4.5 Hypothalamic Subnuclei Analysis

To localize functional alterations within hypothalamic subdivisions, the human hypothalamic atlas of Jensen et al. was applied [18]. Voxel‐wise t‐values from the RCI vs. control contrast (HC − Patient) were intersected with masks of seven functional subnuclei: paraventricular nucleus (PVN), medial preoptic nucleus (MPO), supraoptic nucleus superior compartment (SOSC), mammillary nuclei (MM), ventromedial (VM), dorsomedial (DM), and posterior hypothalamus (PH). For each subnucleus, the proportion of voxels surpassing |t| > 1.994 (uncorrected *p* < 0.05) was computed, and the within‐mask t‐value distribution was visualized as violin plots with the threshold t = −1.994 indicated.

### 4.6 Imaging Transcriptomics Analysis

Microarray gene expression data from all six donors of the Allen Human Brain Atlas (AHBA) were spatially mapped to 55 left‐hemisphere gray‐matter ROIs from the JHU atlas using the abagen toolbox (v0.1.3) [38]; white‐matter ROIs and right‐hemisphere ROIs were excluded because AHBA microarray sampling is restricted primarily to left‐hemisphere gray‐matter compartments. Probes were selected by RNA‐seq–based matching, expression values were normalized within and across donors using the scaled robust sigmoid method, and samples were aggregated within donors before being averaged across donors, yielding a 55 × N_gene region‐by‐gene matrix. Genes with zero variance across ROIs were removed.

ROI‐level T‐statistics were obtained by averaging non‐zero voxel T‐values from the RCI vs. HC contrast within each ROI. For each gene, a Spearman correlation was computed between the ROI‐level T‐vector and ROI‐level gene expression. To control for spatial autocorrelation among neighboring ROIs, 5000 BrainSMASH surrogate T‐maps preserving the empirical variogram were generated [39], and Spearman correlations were recomputed for each surrogate to construct the null distribution.

KEGG pathway enrichment was performed against the KEGG_2021_Human library; pathways with fewer than 15 matched genes were excluded. Weighted gene set enrichment analysis (GSEA) was performed on the full ranked list of all retained genes, with the same enrichment procedure applied to each surrogate‐derived ranking to build the pathway‐level null distribution. Spatially corrected two‐sided *p*‐values were derived from this null distribution, and normalized enrichment scores (NES) were computed against the same‐sign surrogate ES distribution. Benjamini–Hochberg FDR correction was applied separately within positively and negatively enriched pathways; pathways with FDR q < 0.05 were considered significantly enriched.

### 4.7 Animal

All animal procedures were reviewed and approved by the Laboratory Animal Ethics Committee of Xiangya Hospital, Central South University (approval no. 2022020179).

Mice were housed under specific‐pathogen‐free conditions on a 12 h:12 h light/dark cycle (lights on 07:00) at 22°C ± 1°C, with ad libitum access to food and water. Adult male C57BL/6J mice (8 weeks old, ≈ 22–25 g) were used in all experiments to control for sex‐related variability; sex‐specific effects warrant further investigation. The following genetically modified strains were used:

(A) Wild‐type C57BL/6J — Department of Laboratory Animal Science, Central South University.

(B) TRAP2 mice (Fos2A‐iCreERT2;Ai14) [40], generated by crossing Fos2A‐iCreERT2 (Jax #030323) with Rosa26‐LSL‐tdTomato/Ai14 reporter mice (Jax #007914), used for activity‐dependent labeling.

(C) Th‐Cre transgenic mice (kindly provided by Prof. Ren Xu, Xiamen University).

(D) Th‐Cre;Rosa26‐LSL‐iDTR‐tdTomato mice (generated by crossing Th‐Cre with Rosa26‐LSL‐iDTR‐tdTomato; Shanghai Model Organisms Center) used for selective ablation of TH‐positive sympathetic neurons.

All animal experiments were designed and reported in accordance with the ARRIVE 2.0 guidelines (https://arriveguidelines.org/).

### 4.8 RCI Mouse Model Construction

Eight‐week‐old male mice underwent unilateral left‐side supraspinatus tendon detachment‐and‐repair surgery under isoflurane anesthesia (3% induction, 1.5% maintenance), following the protocol described by Bell et al. and adapted in our previous work [41, 42]. Briefly, after fur removal and aseptic preparation, the supraspinatus tendon was exposed, sharply detached from its insertion at the greater tuberosity, and the bony footprint lightly debrided. The tendon was then reattached using a transosseous suture (5‐0 Prolene) through a bone tunnel in the greater tuberosity. Skin was closed with 6‐0 nylon sutures, and mice received subcutaneous buprenorphine (0.05 mg/kg) for post‐operative analgesia (the detailed surgical procedure of RCI model is schematized in Figure S5).

### 4.9 Behavioral Tests

Behavioral assessment used independent, non‐repeated cohorts. All mice undergoing behavioral phenotyping were subjected to RCI surgery on the same day in a single operative batch (n = 54 injured mice, allocated 18 per post‐injury interval: 2wks, 4wks, and 8wks pi), together with a concurrently housed uninjured control group. Each animal completed the open field test and elevated plus maze once only, at its assigned time point; no animal was tested more than once. Where subsequent terminal assays required additional animals, supplementary animals were generated in later operative batches and used exclusively for tissue‐based endpoints; these animals did not undergo behavioral testing and are not included in the behavioral datasets shown in Figure 2A,B.

All assessments were conducted between 09:00 and 12:00 under controlled environmental conditions (50 lux, < 50 dB background noise, 22°C ± 1°C). Experimenters were blinded to group identity during all behavioral data acquisition and analysis.

Open Field Test (OFT). Mice were placed in the center of a 50 × 40 × 40 cm arena and allowed to explore for 10 min. Total distance traveled (cm) and percentage of time spent in the central zone (25 × 20 cm) were quantified using SMART V3.0 software (Panlab). The arena was wiped with 70% ethanol between trials.

Elevated Plus Maze (EPM). The maze comprised two open arms (35 × 5 cm, no walls) and two closed arms (35 × 5 cm, 15‐cm‐high walls) joined by a 5 × 5 cm central platform, elevated 50 cm above the floor. Each mouse was placed in the center facing an open arm and recorded for 5 min. The number of entries into open arms and percentage of time in open arms were quantified using SMART V3.0. The maze was wiped with 70% ethanol between trials.

### 4.10 TRAP2 Whole‐Brain Activity Mapping With Light‐Sheet Microscopy

TRAP2 (Fos2A‐iCreERT2;Ai14) mice received three consecutive daily intraperitoneal injections of 4‐hydroxytamoxifen (4‐OHT, 50 mg/kg/day in corn oil) starting at 4 wks pi, permanently labeling neurons active during this 3‐day window with tdTomato. Throughout the 4‐OHT administration period and the subsequent labeling window, mice remained in their home cages under standard, undisturbed housing conditions, without exposure to additional experimental manipulation. Three days after the final 4‐OHT injection, mice were transcardially perfused with cold PBS followed by 4% PFA.

Whole brains were post‐fixed in 4% PFA overnight, then optically cleared using the SOLID protocol [43], comprising gradient delipidation/dehydration followed by refractive‐index (RI) matching. Samples underwent stepwise dehydration in 30%, 50%, 70%, and 90% (v/v) 1,2‐hexanediol (1,2‐HxD; Macklin), each containing 2% (v/v) N‐butyldiethanolamine (Tokyo Chemical Industry, B0725), followed by thorough dehydration in HxD‐TBN solution (90% v/v 1,2‐HxD / 10% v/v tert‐butanol, with 2% v/v N‐butyldiethanolamine and dH_2_O); for whole adult mouse brains, the five steps were carried out for 1, 0.5, 0.5, 0.5, and 1 d, respectively, at 30°C under gentle shaking. Samples were then immersed in BBPN RI‐matching solution (75% v/v benzyl benzoate, 20% v/v PEGMMA500, 5% v/v N‐butyldiethanolamine) until fully transparent. Native tdTomato fluorescence was used for downstream imaging; no antibody amplification was performed.

Cleared brains were imaged on an UltraMicroscope Blaze light‐sheet microscope (Miltenyi Biotec, Germany) with a 4× objective and 5 µm z‐step, using 561 nm excitation for tdTomato detection. Image volumes were registered to the Allen Mouse Brain Common Coordinate Framework (CCFv3) using ClearMap 2.0 [44]. Fos^TRAP^ (tdTomato^+^) cells were detected by spot‐finding, and spot density (density of detected tdTomato^+^ fluorescent foci, × 10^3^/mm^3^) was quantified per hypothalamic subnucleus as a proxy for relative TRAP‐labeled signal abundance. Group differences for each subnucleus were tested by two‐tailed Student's *t*‐test. Three‐dimensional point clouds of PVN tdTomato^+^ neurons were rendered in Imaris v9.7 to visualize medial/posterior vs. anterior PVN distribution. n = 3 per group.

### 4.11 In Vivo PVN Local Field Potential Recording

Mice were anesthetized with isoflurane (3% induction, 1.5% maintenance) and placed in a stereotaxic frame on a heating pad maintaining body temperature at 37°C. A recording electrode (4‐channel microwire array, KD‐MWA‐4, Kedou) was advanced into the PVN (AP −0.76 mm, ML ±0.26 mm, DV −4.74 mm from dura; Paxinos & Franklin, fourth ed., 2013). Signals were acquired with a multi‐channel in vivo electrophysiology system (Blackrock Microsystems) at 2 kHz. A band‐stop (notch) filter removed 50 Hz line noise and its harmonics, and signals were band‐pass filtered (0.5–300 Hz) to extract the LFP component. For each animal a 60‐s epoch of stable signal was analyzed; power spectral density was computed by Welch's method (Hamming window, nperseg = 4096, 50% overlap), and integrated power quantified within delta (1–4 Hz), theta (4–8 Hz), alpha (8–13 Hz), beta (13–30 Hz), low gamma (30–50 Hz), high gamma (50–100 Hz), and broadband (1–100 Hz) ranges. Recordings were obtained at Control, 2wks, 4wks, and 8wks pi (n = 6 for each group).

### 4.12 PVN Single‐Neuron Connectome Reanalysis

To identify PVN projection‐defined neuronal subtypes, single‐neuron morphological reconstructions and projection metadata from the public dataset of Jiao et al. were obtained [20]. A total of 574 PVN neurons with whole‐brain axon reconstruction were retained after quality control.

Projection‐pattern–based clustering was performed de novo in the present study. For each neuron, axonal projection to 50 standardized target regions across eight anatomical divisions (cortex, cerebral nuclei, thalamus, hypothalamus, midbrain, pons, medulla, and spinal cord; full list in Table S5), was determined from the SWC reconstruction. The 49 brain‐region targets, together with the spinal cord segmentation, constitute the 50 targets and correspond to the projection‐target parcellation of the source dataset. Spinal‐cord‐projecting neurons (SC_Proj subset) were defined separately as those exhibiting reconstructed axon length > 13 200 µm in the spinal cord segmentation.

K‐medoids clustering (Manhattan distance, k = 5 selected by silhouette analysis maximization across k = 2–10) was applied to the projection‐length matrix to identify neuronal subpopulations. Soma coordinates and neuropeptide genotype labels were taken from the original data. Soma coordinates were registered to the Allen CCFv3 template to assess cluster‐specific distribution.

### 4.13 Retrograde Trans‐Synaptic Tracing

Pseudorabies virus (PRV‐CAG‐EGFP and PRV‐CAG‐mRFP, Bartha‐derived; titer 2 × 10^9^ PFU/mL; BrainVTA, Wuhan, China) was used for polysynaptic retrograde tracing. Under isoflurane anesthesia, the supraspinatus enthesis was exposed, and 1 µL of virus was injected into the subchondral bone area of the supraspinatus enthesis as a single deposit using a Hamilton 5 µL microsyringe (model 7633‐01) fitted with a 33‐gauge microinjection needle (model 7803–05; Hamilton, USA) at 0.1 µL/min. Continuous stereomicroscopic monitoring excluded extravasation into the overlying tendon, peri‐articular muscle, or cutaneous tissues. No reflux was observed at the needle track in any animal. The needle was held in place for 30 s post‐injection. Mice were euthanized 5 days post‐injection — a time point at which PRV‐Bartha labeling typically reflects second‐ to third‐order presynaptic neurons relative to the injection site, while mortality risk from PRV‐induced encephalopathy precludes longer survival. Brains, cervico‐thoracic spinal cord, and the sympathetic chain (SCG, stellate ganglion, T2 ganglion) were harvested for immunofluorescence and spatial transcriptomic analysis.

All PRV‐related procedures were conducted in a BSL‐2 facility in accordance with the institutional biosafety guidelines of Central South University.

### 4.14 Spatial Transcriptomic Sequencing and Analysis

#### 4.14.1 Sample Preparation

Brains harvested 5 days post‐PRV injection were fresh‐frozen in OCT, and 10 µm coronal sections at the mPVN level were cryosectioned and mounted on Stereo‐seq capture chips (BGI) [45]. Tissue permeabilization, in situ reverse transcription, second‐strand synthesis, library construction, and sequencing on the BGI DNBSEQ platform were performed using the manufacturer's Stereo‐seq workflow.

#### 4.14.2 Bioinformatic Processing

Raw reads were processed with the Stereo‐seq Analysis Workflow (SAW v5.4) [46]. To enable detection of viral transcripts, the PRV‐Bartha genome sequence and the exogenous EGFP cassette were appended to the mm10 mouse reference FASTA, with corresponding entries added to the GTF annotation, and a STAR genome index was built using saw makeRef (SAW's reference‐building utility) and supplied to SAW for alignment and gene‐by‐spot matrix generation. Quality‐controlled gene‐by‐spot matrices were imported into Stereopy v1.12.0 (Python 3.9) for log‐normalization, highly variable gene selection, dimensionality reduction (PCA → UMAP), and Leiden unsupervised clustering, identifying 11 spatial clusters. Cluster 2 was annotated as the mPVN based on marker‐gene expression and anatomical correspondence with the H&E section.

#### 4.14.3 PRV–Host Gene Correlation

Across all spatial spots, Spearman correlations were computed between total PRV transcript abundance and the expression of each host gene (volcano plot of −log_10_(P_FDR_) vs. Spearman ρ). To complement the continuous correlation analysis with a discrete‐group comparison, a stringent binary classification was applied: spots with total PRV transcript abundance exceeding the 95th percentile of the sample‐wide distribution were designated PRV‐infected. Differences in *Oxt* expression between PRV‐infected and PRV‐uninfected spots were tested using the two‐sided Mann–Whitney U test.

#### 4.14.4 Pattern‐Recognition Co‐Expression Analysis

Spatial co‐expression modules in PRV‐enriched spots were identified using SpaGene with default parameters [47]. Pattern 6, defined by joint elevation of PRV transcripts and *Oxt*, was identified and visualized.

#### 4.14.5 Visualization

Spatial co‐localization of PRV transcripts and *Oxt*‐expressing spots was visualized in Scanpy. Combined immunofluorescence (PRV‐CAG‐mRFP × OXT) on adjacent sections confirmed cellular co‐localization (see Immunofluorescence).

### 4.15 Stereotaxic AAV Injection and Chemogenetic Manipulation

Coordinates (Paxinos & Franklin, fourth ed., 2013): PVN: AP −0.76 mm, ML ± 0.26 mm, DV −4.74 mm from dura.

Under isoflurane anesthesia (3% induction, 1.5% maintenance), 8‐week‐old male mice were placed in a stereotaxic frame. After scalp disinfection (70% ethanol) and midline incision, a 0.5 mm cranial window was drilled at the target coordinates. Viral constructs (BrainVTA Co., Ltd.) were delivered bilaterally at 0.1 µL/min using a microinjection system (WPI): Chemogenetic inhibition (DREADD‐Gi), 150 nL rAAV2/9‐mOXTpr‐hM4D(Gi)‐EGFP (5.71 × 10^1^ ^2^ vg/mL); Chemogenetic activation (DREADD‐Gq), 300 nL rAAV2/9‐mOXTpr‐hM3D(Gq)‐mCherry (2.51 × 10^1^ ^2^ vg/mL); Control, 300 nL rAAV2/9‐mOXTpr‐EGFP (2.51 × 10^1^ ^2^ vg/mL) without DREADD coding sequence.

The mOXT promoter restricts transgene expression to oxytocinergic neurons. Needles were held in place for 10 min after injection, then withdrawn slowly. Mice recovered for at least 3 weeks before further procedures.

CNO administration. Three weeks after AAV injection, clozapine N‐oxide (CNO, 5 mg/kg/day in drinking water) was provided to DREADD‐Gq, DREADD‐Gi, and Control groups. Dosing was calculated assuming a daily intake of 5 mL per ∼ 25 g mouse. After 1 week of CNO pre‐treatment, RCI surgery was performed, and CNO administration was continued throughout the 4‐week post‐injury period until tissue harvest.

DREADDs expression validation. After all experiments, viral expression was confirmed by immunostaining for mCherry/EGFP and OXT (anti‐OXT, Abcam ab212193, 1:400). The proportion of OXT‐immunoreactive PVN neurons co‐expressing the DREADD reporter was quantified across ≥ 3 sections per animal in ImageJ (n = 6 animals per construct). Transduction specificity was 89.68% ± 3.97% for Gq‐DREADDs and 90.82% ± 4.24% for Gi‐DREADDs (mean ± SD; Welch's *t*‐test, *p* = 0.64), with the lowest individual animal at 82.95%. No animal was excluded on the basis of transduction specificity or injection‐site placement.

### 4.16 Selective SCG Sympathetic Neuron Ablation

Th‐Cre;Rosa26‐LSL‐iDTR‐tdTomato mice were assigned to four rotator cuff injury groups: Control, DREADD‐Gi, DREADD‐Gi+SCG_Abl, and SCG_Abl. The DREADD‐Gi and DREADD‐Gi+SCG_Abl groups received bilateral PVN injection of the Gi‐DREADD vector described above; the Control and SCG_Abl groups received the control vector. To ablate TH‐positive sympathetic neurons, diphtheria toxin (DT; Sigma D0564, 100 ng in 2 µL sterile saline) was injected into the left SCG under microscopic guidance in the DREADD‐Gi+SCG_Abl and SCG_Abl groups. The two non‐ablated groups received an equal volume of sterile saline at the same site. CNO administration and rotator cuff injury modeling followed the procedures described above. Ablation was assessed by quantifying the loss of tdTomato fluorescence in SCG sections using ImageJ (n = 6 per group, for each quantitative outcome in Figure 6C–F).

### 4.17 In Vivo SCG Local Field Potential Recording

The SCG was selected for electrophysiological interrogation and for the subsequent ablation experiments because it was the most robustly and consistently labeled ganglion in our retrograde tracing experiments, and because it is surgically accessible for both local ablation and microelectrode implantation, avoiding the respiratory motion artefact that complicates thoracic ganglion recordings.

Mice were anesthetized with 1.5% isoflurane and placed supine on a heating pad to maintain body temperature at 37°C. A midline cervical incision was made, and the left common carotid artery (CCA) and vagus nerve were carefully retracted to expose the carotid bifurcation. The SCG was identified posterior to the bifurcation and dissected free of surrounding adipose tissue and fascial attachments under a stereomicroscope. A tungsten microelectrode (Beijing Kesheng Technology, 38.1 µm shaft diameter) was advanced into the SCG parenchyma using a manual micromanipulator.

Local field potential (LFP) signals were recorded using a multi‐channel in vivo electrophysiology acquisition system (Blackrock Microsystems, Cereplex Direct, 128‐channel) at a sampling rate of 2 kHz. Raw signals were imported into MATLAB R2018b (Signal Analyzer toolbox); a band‐stop (notch) filter was applied to remove 50 Hz line noise and its harmonics, followed by a 500 Hz low‐pass filter to extract the LFP component for downstream analysis. Firing rate (Hz) was computed from threshold‐crossing time points. Recordings were performed at 4 weeks post‐injury for Control, DREADD‐Gi, and DREADD‐Gq groups (n = 6 per group).

### 4.18 SCG LFP Signal Analysis

All offline analyses were performed in MATLAB R2021a and Python 3.9. For each animal, a 10‐s segment of stable signal was extracted. Welch power spectral density (PSD) was computed using a Hamming window (nperseg = 2048, 50% overlap), with identical parameters applied across all downstream spectral analyses.

Canonical Discriminant Analysis (CDA). To explore which spectral components carried group‐discriminative information in a data‐driven manner, log‐transformed and z‐scored PSD values were submitted to a linear discriminant analysis (Spectral LDA) across the three groups; per‐frequency discriminative weights, derived from the absolute standardized canonical coefficients, were aggregated over a 5‐Hz sliding window, and the top‐ranked, adjacency‐merged windows yielded a candidate band. Given the feature dimensionality and the absence of out‐of‐sample validation, this analysis is presented as an exploratory feature‐selection step rather than a confirmatory test on individual frequency bands.

Broadband high‐frequency LFP power (>50 Hz, predominantly the 60–200 Hz “high‐gamma” range) is a well‐established electrophysiological index of local multi‐unit spiking activity in the vicinity of the recording electrode [48, 49, 50]. We applied this principle to SCG LFP recordings as an index of local sympathetic firing. The validity of this index in the SCG is supported by the concordant changes between 60–200 Hz integrated power and independently quantified spike‐level burst metrics (Figure 5H). Integrated power within 60–200 Hz was compared across groups by one‐way ANOVA with Tukey's post‐hoc test.

Raw signal spikes were detected using a threshold set at four times the median absolute deviation (MAD) of the baseline noise. Spike waveforms were sorted in Plexon Offline Sorter v4 using principal component analysis (PCA)‐based clustering followed by manual refinement. Only well‐isolated units with an L‐ratio < 0.1 and < 1% refractory‐period violations were included. Bursts were defined as sequences of at least three spikes with consecutive interspike intervals < 100 ms. Burst rate (Hz) and the mean number of spikes per burst were compared among groups.

### 4.19 Immunofluorescence

Mice were transcardially perfused with cold PBS followed by 4% PFA. Brains were post‐fixed overnight at 4°C, cryoprotected through graded sucrose (10%, 20%, 30%), embedded in OCT, and cryosectioned coronally at 10 µm (Leica CM1950). SCG samples were processed similarly at 8 µm.

Sections were blocked in 5% donkey serum / 0.3% Triton X‐100 in PBS for 1 h at room temperature, then incubated overnight at 4°C with primary antibodies: anti‐Oxytocin (Abcam ab212193, 1:400), anti‐cFos (Synaptic Systems, #226017), anti‐Tyrosine Hydroxylase (TH; Millipore, #HPA061003, 1:500).

Alexa Fluor 488 / 594 / 647‐conjugated donkey secondary antibodies (Abcam, 1:500) were applied for 2 h at room temperature in the dark, with DAPI counterstaining. Z‐stacks (0.5 µm steps) were acquired on a Zeiss AxioImager.M2 equipped with the Apotome.2 system (Zeiss, Solms, Germany).

Quantification. SCG c‐Fos^+^/TH^+^ fluorescence‐intensity ratios were computed in ImageJ (Fiji) on background‐subtracted images across ≥ 3 sections per animal, with the experimenter blinded to group assignment. PVN PRV–neuropeptide co‐localization was scored bidirectionally across ≥ 3 sections per animal (n = 6 animals per marker): the proportion of OXT‐, AVP‐, or CRH‐immunoreactive cells co‐expressing PRV signal, and reciprocally the proportion of PRV‐labeled cells expressing each marker.

### 4.20 ELISA

Sample collection was standardized at 11:00 AM to minimize circadian variability (n = 6 per group). Whole blood was collected via the orbital venous plexus under terminal anesthesia, allowed to clot at room temperature for 2–3 h, and centrifuged at 3000 g for 20 min at 4°C; serum was aliquoted and stored at −80°C.

For tissue ELISA, BTI tissue or PVN‐containing tissue (microdissected from 1200 µm‐thick coronal slices at the PVN level using a 3 mm tissue punch in accordance with Paxinos & Franklin, fourth ed., 2013) was rinsed in cold PBS, weighed, and homogenized in PBS (0.01 M, pH 7.4) containing protease inhibitors using a glass homogenizer on ice. Homogenates were centrifuged at 12 000 rpm for 10 min at 4°C, and supernatants were assayed.

NE was measured with the NE ELISA kit (E‐EL‐0047, Elabscience); oxytocin (OXT) was measured with the OXT ELISA kit (E‐EL‐0029, Elabscience) according to the manufacturer's instructions. Total protein in tissue homogenates was measured by BCA assay (Beyotime P0010). NE concentrations in BTI homogenates were expressed as ng/mL by ELISA.

### 4.21 Micro‐CT Analysis

Fresh BTI specimens (n = 6 per group) were fixed briefly in 4% PFA and scanned with a U‐CT Micro‐CT system (MILabs, Netherlands) at 9 µm isotropic voxel size, 50 kV, 200 µA, with 0.5 mm aluminum filtration. Reconstructions were analyzed in Imalytics Preclinical software. A standardized region of interest in the greater tuberosity adjacent to the supraspinatus footprint was segmented, and the following microarchitectural parameters were computed: bone volume fraction (BV/TV, %), trabecular thickness (Tb.Th, mm), trabecular number (Tb.N, 1/mm), and trabecular separation (Tb.Sp, mm).

### 4.22 Biomechanical Testing

After Micro‐CT, specimens (n = 6 per group) were thawed in PBS at room temperature, and all soft tissues except the supraspinatus muscle, tendon, and humerus were carefully removed. The supraspinatus–humerus complex was clamped in a custom fixture with the humerus mounted in dental cement and the supraspinatus tendon held in pneumatic grips on an MTS Insight 23 system equipped with a 100 N load cell. After preconditioning at 0.05 N for 1 min, tendon thickness and width were measured with a digital caliper to compute cross‐sectional area (mm^2^). Specimens were elongated to failure at 0.03 mm/s. The following parameters were extracted from load–displacement curves: failure load (N), stiffness (N/mm; slope of the linear portion), and ultimate tensile strength (MPa; failure load/cross‐sectional area).

### 4.23 Histological Evaluation

BTI specimens (n = 6 per group) were fixed in 4% PFA, decalcified in 10% EDTA for 2 weeks, dehydrated through a graded ethanol series (70%, 80%, 90%, each 3 h), cleared in n‐butanol for 6 h, and paraffin‐embedded. Sections (5 µm) were stained with hematoxylin and eosin (H&E) and toluidine blue (for proteoglycan visualization in fibrocartilage) according to standard protocols. BTI healing quality was scored using a modified Ide scoring system (15 = best; 0 = worst) [51], evaluating fibrocartilage organization, cellular alignment, tidemark formation, and fiber continuity, by two observers blinded to group assignment; discrepancies were resolved by consensus.

### 4.24 Quantitative Real‐Time PCR

At 4 weeks post‐injury, BTI specimens were snap‐frozen in liquid nitrogen, homogenized in TRIzol (Invitrogen), and total RNA extracted; only samples with A260/A280> 1.8 were retained. cDNA was synthesized using the GoScript Reverse Transcription Kit (Promega). qPCR was performed with SYBR Green mix (Takara) on a QuantStudio 3 system (Applied Biosystems) using primer sequences for *Aggrecan*, *Sox9*, *Ocn*, *Runx2*, and *β‐actin* (housekeeping). Cycling: 95°C × 30 s; 40 cycles of 95°C × 5 s and 60°C × 30 s; melting‐curve analysis 60°C–95°C. Relative expression was calculated using the 2^−ΔΔCt^ method, normalized to β‐actin and to the Control group.

### 4.25 Statistical Analysis

Data were analyzed in RStudio (R 4.2), GraphPad Prism 9.0, and SPSS 26.0. Data pre‐processing, including normalization and the criteria used to evaluate and exclude outlying data, is described in the corresponding Methods subsections. Distribution normality was assessed using the Shapiro–Wilk test. Two‐group comparisons of normally distributed data used Welch's two‐sample *t*‐test (unpaired, two‐tailed); non‐normal data used the Mann–Whitney U test. Multi‐group comparisons used one‐way ANOVA followed by Tukey's HSD post‐hoc test, or Kruskal–Wallis followed by Dunn's post‐hoc test when normality was violated. For high‐dimensional contexts (ROI zALFF, gene–t correlations, KEGG pathways), Benjamini–Hochberg FDR correction was applied (q < 0.05 considered significant). For correlational analyses, Spearman's rank correlation coefficient was used. All *p*‐values are two‐sided. Significance levels in figures: ^*^ *p* < 0.05, ^**^ *p* < 0.01, ^***^ *p* < 0.001, ^****^ *p* < 0.0001, ns = not significant. Data are presented as mean ± SEM unless otherwise specified. Sample sizes (n) for each panel are detailed in the corresponding figure legends.

## Author Contributions

**Liyang Wan**: conceptualization, methodology, data curation, writing – original draft, writing – review and editing. **Tingmo Huang**: methodology, data curation, writing – original draft. **Liyue Zeng**: data collection. **Ruixue Du**: methodology. **Tao Zhang**: review and editing. **Hengshuo Zhang**: methodology. **Shen Liu**: data collection. **Jianzhong Hu**: review and editing, project administration. **Hongbin Lu**: investigation, writing – review and editing, funding acquisition, project administration.

## Funding

This study was supported by the National Natural Science Foundation of China (No. 82230085 and 82272572) and the Hunan Provincial Natural Science Foundation (No. 2025JJ60682).

## Ethics Statement

The clinical study was reviewed and approved by the Medical Ethics Committee of Xiangya Hospital, Central South University (approval no. 202307601), and was conducted in accordance with the Declaration of Helsinki. All participants provided written informed consent prior to enrolment. All animal procedures were reviewed and approved by the Laboratory Animal Ethics Committee of Xiangya Hospital, Central South University (approval no. 2022020179), and were performed in accordance with institutional guidelines for the care and use of laboratory animals.

## Conflicts of Interest

The authors declare no conflicts of interest.

## Supporting information




**Supporting File 1**: advs78190‐sup‐0001‐SuppMat.pdf.

**Supporting File 2**: advs78190‐sup‐0002‐SuppMat.zip.

## Data Availability

The raw resting‐state fMRI data cannot be publicly shared due to patient privacy regulations; de‐identified group‐level statistical maps are available from the corresponding author upon reasonable request. Spatial transcriptomics (Stereo‐seq) sequencing data will be deposited prior to publication. The single‐neuron morphological and projection data used for connectome reanalysis are publicly available from Jiao et al. (2025, Nature Neuroscience; https://doi.org/10.1038/s41593‐025‐01919‐0). All other data supporting the findings of this study and analysis code are available from the corresponding author upon reasonable request.
