# Supplementary material for: A Hypothalamic Oxytocinergic–Sympathetic Axis Couples Anxiety Dysregulation With Impaired Tendon–Bone Repair
Source: Adv Sci (Weinh). 2026 Oct 6:e78190. Online ahead of print. doi: 10.1002/advs.78190 (PMC13643106; doi:10.1002/advs.78190)
Supplement: Supplementary file 1 [file ADVS-9999-e78190-s002.pdf]

Supplementary Materials for

A Hypothalamic Oxytocinergic–Sympathetic Axis Couples Anxiety Dysregulation with Impaired Tendon–Bone Repair

Liyang Wan[1,2,3]†, Tingmo Huang[1,2,3]†, Liyue Zeng[1,2,3], Ruixue Du[1,2,3], Hengshuo Zhang[1,2,3], Shen Liu[1,2,3], Tao Zhang[1,2,3], Jianzhong Hu[2,3,4,*], Hongbin Lu[1,2,3,**]

†These authors contributed equally
*Correspondence: jianzhonghu@csu.edu.cn
**Correspondence: hongbinlu@csu.edu.cn

This file includes:
Tables S1 to S8
Figures S1 to S5

Supplementary Table S1.

S1a. Demographic and psychological characteristics (RCI vs HC)

Variable	RCI (n=43)	HC (n=30)	Test	Statistic	p-value
Age (years)	45.93 ± 8.82	43.65 ± 9.48	Welch t	t=1.039	0.3031
Sex (male/female)	19 / 24	14 / 16	Chi-square	χ^2=0.000	1.000
Education (years)	13.53 ± 4.11	14.47 ± 3.65	Mann-Whitney U	U=594.5	0.562
BMI (kg/m²)	23.20 ± 2.68	22.65 ± 2.74	Welch t	t=0.853	0.397
HADS-A (anxiety)	6.07 ± 3.49	3.77 ± 2.62	Mann-Whitney U	U=892.5	0.0053*
HADS-D (depression)	4.19 ± 3.62	2.97 ± 2.53	Mann-Whitney U	U=759.0	0.1992

S1b. Injury-specific clinical characteristics (patient group, n=43)

Variable	Summary	n
Symptom duration (months)	13.67 ± 20.80	43
VAS pain (mm)	47.47 ± 24.15	43
Constant score	61.28 ± 15.10	43
MRI grade	I: 10, II: 22, III: 11	43
Injured side	Right: 25, Left: 18	43

Supplementary Table S1. Demographic and psychological baseline characteristics of the RCI and healthy control cohorts. Related to Fig. 1B. Baseline characteristics of the 43 patients with rotator cuff injury (RCI) and 30 healthy controls (HC) included in all clinical analyses. Continuous variables are reported as group mean ± SD and sex as the male/female count. For each variable the table reports the test applied, test statistic, and the two-tailed p value. Age, sex, education and BMI did not differ between groups, confirming that the two cohorts were demographically matched, whereas HADS-A anxiety scores were significantly elevated in patients and HADS-D depression did not differ. The lower block (Table S1b) summarises injury-specific clinical characteristics available only for the patient group (n = 43)—symptom duration, VAS pain, Constant score, MRI tear grade and injured side—reported as mean ± SD or category counts. *$p < 0.05$.

Supplementary Table S2.

ROI	Anatomical region	HC (mean±SD)	RCI (mean±SD)	t (Welch)	p_raw	p_FDR (Welch)
Jhu183	PIns_R (posterior insula right)	-0.466 ± 0.171	-0.315 ± 0.179	-3.693	0.00046	0.0365*
Jhu068	dorsal_ACC_R (dorsal anterior cingulate gyrus right)	0.185 ± 0.202	0.355 ± 0.290	-3.497	0.00082	0.0365*
Jhu074	Amyg_R (amygdala right)	-0.462 ± 0.190	-0.295 ± 0.219	-3.442	0.001	0.0365*
Jhu086	Hypothalamus_R (hypothalamus right)	0.878 ± 0.855	0.287 ± 0.526	3.405	0.00144	0.0396*
Jhu073	Amyg_L (amygdala left)	-0.340 ± 0.217	-0.145 ± 0.325	-3.237	0.00184	0.0405*
Jhu085	Hypothalamus_L (hypothalamus left)	0.697 ± 0.876	0.145 ± 0.339	3.271	0.00241	0.0442*
Jhu168	Mammillary_R (mammillary body right)	1.248 ± 1.365	0.523 ± 0.696	2.683	0.01049	0.137
Jhu072	Ins_R (insular right)	-0.338 ± 0.215	-0.216 ± 0.197	-2.623	0.01124	0.137
Jhu167	Mammillary_L (mammillary body left)	1.174 ± 1.170	0.541 ± 0.726	2.603	0.01244	0.137
Jhu046	PHG_R (parahippocampal gyrus right)	-0.272 ± 0.187	-0.156 ± 0.197	-2.569	0.01246	0.137

Supplementary Table S2. Whole-brain grey-matter zALFF comparison between RCI patients and healthy controls. Related to Fig. 1C – D. zALFF was compared between the 43 patients with RCI and 30 HC across all 110 grey-matter ROIs (atlas label type 1) of the 189-region Johns Hopkins University (JHU) atlas. For each ROI, age and sex were first regressed out of the zALFF signal (covariate residualisation), and the residualised values were compared by a two-tailed Welch unequal-variance t-test; degrees of freedom were Welch – Satterthwaite corrected and therefore vary across ROIs. Multiple comparisons were controlled by the Benjamini – Hochberg FDR procedure across the 110 ROIs. For each ROI the table reports the JHU index, the anatomical region, the raw (un-residualised) group mean ± SD for HC and RCI, the Welch t value (contrast HC − patient, t > 0 indicates lower zALFF in patients), the uncorrected p value (p_raw), the FDR-corrected p value (p_FDR, Welch). ROIs are sorted by ascending p_raw. Six grey-matter regions reach FDR significance ($q < 0.05$): the bilateral hypothalamus, right posterior insula, bilateral amygdala and right dorsal anterior cingulate cortex. Representative first 10 rows shown; the complete dataset (110 ROIs) is provided as Supplementary File S1_ROI_zALFF.xlsx. *$p < 0.05$.

Supplementary Table S3.

ROI	Anatomical region	Scale	n	Spearman r	r^2	p_raw	p_FDR (n=6)
Jhu068	dorsal_ACC_R (dorsal anterior cingulate gyrus right)	HADS-A	43	0.103	0.011	0.5105	0.613
Jhu068	dorsal_ACC_R (dorsal anterior cingulate gyrus right)	HADS-D	43	0.172	0.03	0.2687	0.322
Jhu073	Amyg_L (amygdala left)	HADS-A	43	0.164	0.027	0.2919	0.553
Jhu073	Amyg_L (amygdala left)	HADS-D	43	0.173	0.03	0.2676	0.322
Jhu074	Amyg_R (amygdala right)	HADS-A	43	0.14	0.02	0.3689	0.553
Jhu074	Amyg_R (amygdala right)	HADS-D	43	0.051	0.003	0.7459	0.746
Jhu085	Hypothalamus_L (hypothalamus left)	HADS-A	43	-0.375	0.14	0.0133	0.04*
Jhu085	Hypothalamus_L (hypothalamus left)	HADS-D	43	-0.388	0.151	0.0101	0.061
Jhu086	Hypothalamus_R (hypothalamus right)	HADS-A	43	-0.422	0.178	0.0049	0.029*
Jhu086	Hypothalamus_R (hypothalamus right)	HADS-D	43	-0.315	0.099	0.0396	0.119
Jhu183	PIns_R (posterior insula right)	HADS-A	43	-0.024	0.001	0.8801	0.88
Jhu183	PIns_R (posterior insula right)	HADS-D	43	-0.259	0.067	0.0941	0.188

Supplementary Table S3. Correlations between regional zALFF and HADS anxiety and depression scores across the six FDR-significant grey-matter regions. Related to Fig. 1E. For each of the six grey-matter regions of interest (ROIs) showing a significant RCI-versus-HC zALFF group difference (Table S2), resting-state zALFF was correlated with individual HADS scores within the 43 patients with RCI. Associations were quantified by Spearman's rank correlation; each ROI is reported twice—once for the anxiety subscale (HADS-A) and once for the depression subscale (HADS-D)—yielding 12 rows. Benjamini–Hochberg FDR correction was applied separately within each subscale across the six ROIs. For each row the table lists the JHU atlas ROI index, the anatomical region, the HADS subscale, the sample size, Spearman's r, the coefficient of determination r^2 , the uncorrected p value (p_raw), and the FDR-corrected p value (p_FDR, n = 6). Whereas Fig. 1E plots only the two hypothalamic ROIs against HADS-A, this table additionally provides the HADS-D associations, enabling assessment of the anxiety-specificity of the hypothalamic findings. *$p < 0.05$.

Supplementary Table S4.

Acronym	Full name	Control mean±SD (spots/mm³)	4wks pi mean±SD (spots/mm³)	p value	Significance	Shown in Fig. 2D
LM	Lateral mammillary nucleus	14856.2 ± 1871.7	5867.6 ± 3123.0	0.0129	*	
PVH	Paraventricular hypothalamic nucleus	11782.3 ± 450.2	6436.3 ± 2712.9	0.0281	*	Yes
TU	Tuberal nucleus	21572.6 ± 3617.8	8306.0 ± 5898.2	0.0294	*	
LHA	Lateral hypothalamic area	25498.6 ± 6171.9	11744.8 ± 4422.0	0.0349	*	
AHN	Anterior hypothalamic nucleus	20279.5 ± 1462.3	13903.2 ± 3472.6	0.0428	*	
PeF	Perifornical nucleus	24683.1 ± 4506.5	11849.9 ± 6648.7	0.0505	†	
TM	Tuberomammillary nucleus	19934.5 ± 3751.6	7571.9 ± 6889.0	0.0525	†	
PMv	Ventral premammillary nucleus	15907.0 ± 2533.1	9333.0 ± 3361.4	0.0538	†	
LPO	Lateral preoptic area	19816.4 ± 2215.0	11803.0 ± 4641.9	0.0542	†	
MPO	Medial preoptic area	25253.1 ± 3709.6	14594.2 ± 5831.5	0.0557	†	Yes
AVPV	Anteroventral periventricular nucleus	6050.4 ± 1489.9	3021.1 ± 1338.5	0.0588	†	
SOSC	Supraoptic nucleus, superior compartment	13192.1 ± 6280.5	5015.6 ± 1096.9	0.0905	†	Yes
ME	Median eminence	932.4 ± 779.9	6330.0 ± 4187.0	0.0932	†	
MPN	Medial preoptic nucleus	25283.1 ± 3582.4	16817.9 ± 6292.6	0.1129	ns	
DMH	Dorsomedial nucleus of the hypothalamus	16521.2 ± 4990.4	9927.0 ± 2950.9	0.1202	ns	Yes
PMd	Dorsal premammillary nucleus	9551.1 ± 611.0	8496.4 ± 813.2	0.1469	ns	
MEPO	Median preoptic nucleus	5008.4 ± 3666.5	1675.1 ± 1583.0	0.2218	ns	
PD	Posterodorsal preoptic nucleus	55854.3 ± 17939.4	31335.9 ± 29561.9	0.2867	ns	

Acronym	Full name	Control mean±SD (spots/mm³)	4wks pi mean±SD (spots/mm³)	p value	Significance	Shown in Fig. 2D
PVa	Periventricular hypothalamic nucleus anterior part	4258.5 ± 4033.6	1316.3 ± 1190.1	0.2923	ns	
AVP	Anteroventral preoptic nucleus	20814.0 ± 13736.2	12109.2 ± 3267.0	0.3458	ns	
PVp	Periventricular hypothalamic nucleus posterior part	3831.9 ± 2928.7	6426.6 ± 3302.8	0.3662	ns	
VMH	Ventromedial hypothalamic nucleus	16049.6 ± 6102.8	11571.0 ± 4668.5	0.3698	ns	Yes
PH	Posterior hypothalamic nucleus	13357.6 ± 2594.7	10732.5 ± 4246.6	0.4126	ns	Yes
ZI	Zona incerta	32006.1 ± 20689.7	21445.6 ± 13149.1	0.497	ns	
PVi	Periventricular hypothalamic nucleus intermediate part	1061.4 ± 316.9	1776.3 ± 1946.1	0.564	ns	
MM	Medial mammillary nucleus	7220.2 ± 2893.7	5761.2 ± 2840.8	0.5669	ns	Yes
STN	Subthalamic nucleus	67344.3 ± 55668.8	45978.2 ± 36201.9	0.607	ns	
ARH	Arcuate hypothalamic nucleus	6009.4 ± 5167.3	5178.4 ± 2370.9	0.8126	ns	

Supplementary Table S4. Fos^{TRAP+} neuron density across hypothalamic nuclei in Control versus 4-weeks-post-injury mice. Related to Fig. 2D. Fos^{TRAP+} (tdTomato+) spot density (spots/mm³) was quantified by TRAP2 light-sheet whole-brain imaging across all 28 hypothalamic nuclei delineated in the Allen CCFv3 (n = 3 mice per group). For each nucleus the table reports group mean±SD, the two-tailed unpaired Student's t-test p value (df = 4), nuclei are sorted by ascending p value. Five nuclei reach significance ($p < 0.05$), none survived Benjamini-Hochberg FDR correction across 28 nuclei (minimum q = 0.150). The "Shown in Fig. 2D" column marks the seven a priori functional subnuclei corresponding to the human hypothalamic atlas used in Fig. 1F. *$p < 0.05$*; †*$p < 0.10$; ns, not significant.*

Supplementary Table S5.

No.	Acronym	Full name	Anatomical division	Allen CCFv3 ID
1	Isocortex	Isocortex	Cortex	315
2	OLF	Olfactory areas	Cortex	698
3	HPF	Hippocampal formation	Cortex	1089
4	ACB	Nucleus accumbens	Cerebral nuclei	56
5	LS	Lateral septal nucleus	Cerebral nuclei	242
6	MEA	Medial amygdalar nucleus	Cerebral nuclei	403
7	CEA	Central amygdalar nucleus	Cerebral nuclei	536
8	BST	Bed nuclei of the stria terminalis	Cerebral nuclei	351
9	SI	Substantia innominata	Cerebral nuclei	342
10	MS	Medial septal nucleus	Cerebral nuclei	564
11	BMA	Basomedial amygdalar nucleus	Cerebral nuclei	319
12	BLA	Basolateral amygdalar nucleus	Cerebral nuclei	295
13	PA	Posterior amygdalar nucleus	Cerebral nuclei	780
14	PVT	Paraventricular nucleus of the thalamus	Thalamus	149
15	LH	Lateral habenula	Thalamus	186
16	MD	Mediodorsal nucleus of thalamus	Thalamus	362
17	PT	Parataenial nucleus	Thalamus	15
18	AM	Anteromedial nucleus	Thalamus	127
19	SPFm	Subparafascicular nucleus magnocellular part	Thalamus	414
20	ARH	Arcuate hypothalamic nucleus	Hypothalamus	223
21	VMH	Ventromedial hypothalamic nucleus	Hypothalamus	693
22	DMH	Dorsomedial nucleus of the hypothalamus	Hypothalamus	830
23	PVH	Paraventricular hypothalamic nucleus	Hypothalamus	38
24	MPO	Medial preoptic area	Hypothalamus	523
25	MPN	Medial preoptic nucleus	Hypothalamus	515
26	AHN	Anterior hypothalamic nucleus	Hypothalamus	88
27	LPO	Lateral preoptic area	Hypothalamus	226
28	TU	Tuberal nucleus	Hypothalamus	614
29	TMv	Tuberomammillary nucleus ventral part	Hypothalamus	1

No.	Acronym	Full name	Anatomical division	Allen CCFv3 ID
30	PMv	Ventral premammillary nucleus	Hypothalamus	1004
31	ZI	Zona incerta	Hypothalamus	797
32	LHA	Lateral hypothalamic area	Hypothalamus	194
33	PH	Posterior hypothalamic nucleus	Hypothalamus	946
34	MM	Medial mammillary nucleus	Hypothalamus	491
35	ME	Median eminence	Hypothalamus	10671
36	PAG	Periaqueductal gray	Midbrain	795
37	SNr	Substantia nigra reticular part	Midbrain	381
38	VTA	Ventral tegmental area	Midbrain	749
39	DR	Dorsal nucleus raphe	Midbrain	872
40	SCm	Superior colliculus motor related	Midbrain	294
41	MRN	Midbrain reticular nucleus	Midbrain	128
42	PB	Parabrachial nucleus	Pons	867
43	PCG	Pontine central gray	Pons	898
44	PRNr	Pontine reticular nucleus	Pons	146
45	MY	Medulla	Medulla	354
46	IO	Inferior olivary complex	Medulla	83
47	NTS	Nucleus of the solitary tract	Medulla	651
48	PARN	Parvicellular reticular nucleus	Medulla	852
49	IRN	Intermediate reticular nucleus	Medulla	136
50	SC	Spinal cord	Spinal cord	

Supplementary Table S5. Whole-brain projection target regions used for K-medoids clustering of PVN projection neurons. Related to Fig. 2I – L. The 50 standardized target regions to which the axonal projection of each PVN neuron was scored. Regions 1 – 49 correspond to the projection-target parcellation provided in the source single-neuron projectome dataset (Jiao et al., 2025); no additional target selection was applied. The spinal cord (region 50) was scored separately in the present study. For each region the table lists the acronym, full structure name, anatomical division, Allen Mouse Brain CCFv3 structure ID. Spinal-cord projection was scored using an axon-length threshold of 13,200 μm.

Supplementary Table S6.

Gene	ρ (rho)	r^2	n spots	p_raw	p_FDR
Oxt	0.160	0.026	7189	1.60×10^{-42}	1.71×10^{-39}
Ly6c2	0.144	0.021	7189	1.53×10^{-34}	1.55×10^{-31}
Ccl2	0.138	0.019	7189	6.55×10^{-32}	5.85×10^{-29}
Acod1	0.132	0.017	7189	2.54×10^{-29}	2.11×10^{-26}
Crh	0.127	0.016	7189	2.91×10^{-27}	2.17×10^{-24}
Slfn4	0.127	0.016	7189	4.06×10^{-27}	2.93×10^{-24}
H2-D1	0.125	0.016	7189	1.28×10^{-26}	8.93×10^{-24}
Arhgap30	0.119	0.014	7189	5.38×10^{-24}	3.65×10^{-21}
AA467197	0.117	0.014	7189	2.77×10^{-23}	1.77×10^{-20}
Ccl3	0.110	0.012	7189	7.86×10^{-21}	4.50×10^{-18}

Supplementary Table S6. Genome-wide Spearman correlation between host gene expression and PRV transcript abundance across spatial spots. Related to Fig. 3G. Across all spatial spots of the mPVN Stereo-seq section, total pseudorabies virus (PRV) transcript abundance—computed as the summed raw counts of 31 PRV genes—was correlated with the log-normalized expression of every host gene by Spearman's rank correlation. Multiple comparisons were controlled by the Benjamini–Hochberg FDR procedure across all tested genes. For each gene the table lists the gene symbol, Spearman's ρ (rho), the coefficient of determination r^2, the number of spots, the uncorrected p value (p_raw), the FDR-corrected p value (p_FDR). Genes are sorted by ascending p_FDR, with Oxt emerging as the most strongly and significantly positively correlated host gene. Representative top 10 host genes (non-PRV, non-interferon) sorted by ascending p_FDR are shown; the complete genome-wide dataset (22,348 genes) is provided as Supplementary File S2_PRV_host_spearman.csv.

Supplementary Table S7.

Neuropeptide	n PRVhigh dominant	% of assigned	% of all PRVhigh	n PRVhigh expressing	% expressing	Total PRVhigh spots	n assigned spots
Oxt	131	55.98	36.49	161	44.85	359	234
Avp	48	20.51	13.37	81	22.56	359	234
Trh	17	7.26	4.74	38	10.58	359	234
Sst	15	6.41	4.18	47	13.09	359	234
Tac1	10	4.27	2.79	14	3.9	359	234
Crh	8	3.42	2.23	26	7.24	359	234
Penk	4	1.71	1.11	7	1.95	359	234
Pdyn	1	0.43	0.28	6	1.67	359	234
Adcyap1	0	0.0	0.0	0	0.0	359	234

Supplementary Table S7. Neuropeptidergic composition of PRV high-transcript spatial spots. Related to the upper bar graph in Fig. 3H. PRV high-transcript ("PRV-high") spots were defined as spatial spots whose total PRV transcript abundance exceeded the 95th percentile of the section-wide distribution. Within these spots, the neuropeptidergic identity of each spot was assigned to the most highly expressed of nine PVN neuropeptide markers (Oxt, Avp, Sst, Trh, Crh, Penk, Adcyap1, Tac1, and Pdyn) using log-normalized expression. For each marker the table reports the gene symbol, the number of PRV-high spots for which it is the dominant neuropeptide, its proportion among assigned spots and among all PRV-high spots, the number and proportion of PRV-high spots in which the gene is detectably expressed, and the total number of PRV-high and assigned spots; rows are sorted by descending dominant proportion. This table provides the numerical values and sample sizes underlying the neuropeptide-composition bar graph in Fig. 3H, in which Oxt constitutes the largest fraction, followed by Avp and Sst.

Supplementary Table S8.

Marker	PRV+ cells	Marker+ cells	Colocalized cells	Colocalized / Marker+ (%)	Colocalized / PRV+ (%)
OXT	81	65	43	66.15	53.09
OXT	74	61	35	57.38	47.30
OXT	92	70	43	61.43	46.74
OXT	68	59	33	55.93	48.53
OXT	56	67	32	47.76	57.14
OXT	77	63	37	58.73	48.05
AVP	82	55	21	38.18	25.61
AVP	75	88	18	20.45	24.00
AVP	94	76	16	21.05	17.02
AVP	68	79	14	17.72	20.59
AVP	86	91	19	20.88	22.09
AVP	71	84	15	17.86	21.13
CRH	58	92	8	8.70	13.79
CRH	85	72	11	15.28	12.94
CRH	97	83	9	10.84	9.28
CRH	62	58	9	15.52	14.52
CRH	78	67	6	8.96	7.69
CRH	106	88	14	15.91	13.21

Supplementary Table S8. Raw per-animal counts underlying the bidirectional PRV/neuropeptide colocalization analysis. Related to Fig. 3J. PRV+ denotes the number of PRV-labeled neurons recorded in the marker-specific PVN sections included in the colocalization analysis, Marker+ the number of marker-immunoreactive neurons in the same sampled sections, and Colocalized the double-positive count. Each marker was quantified in a separate staining set; PRV+ denominators are therefore marker-specific and need not be identical across OXT, AVP, and CRH datasets. Percentages were calculated as Colocalized/Marker+ x 100 and Colocalized/PRV+ x 100. Across six animals per marker (mean +/- SD), the respective percentages were OXT: 57.90 +/- 6.14% and 50.14 +/- 4.11%; AVP: 22.69 +/- 7.73% and 21.74 +/- 2.97%; CRH: 12.53 +/- 3.41% and 11.90 +/- 2.75%(n = 6 mice per group).

Supplementary Figure S1.

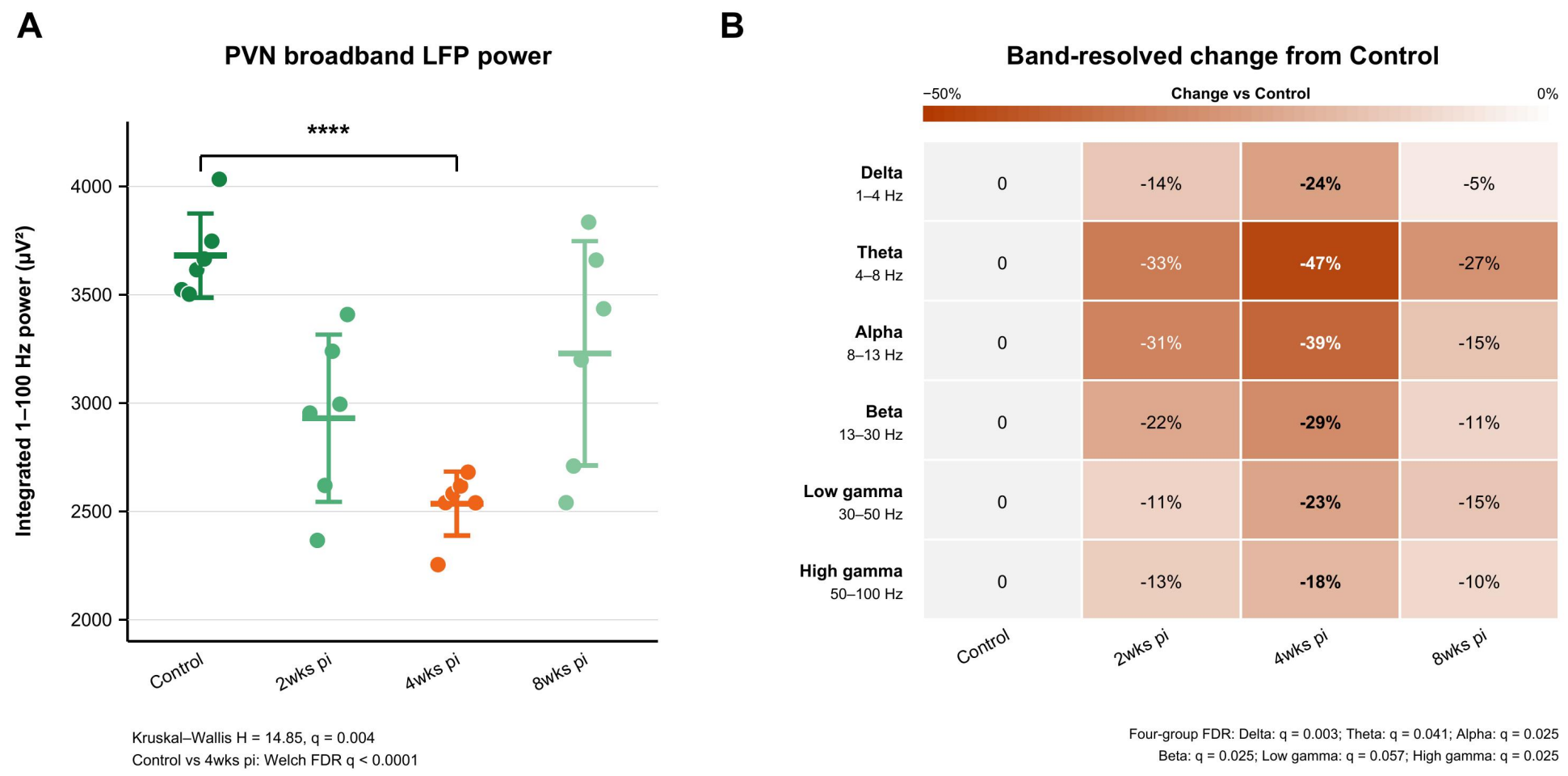

Supplementary Figure S1. Full post-injury time course of PVN local field potential (LFP) power. Related to Fig. 2F-H. Recordings were obtained from Control, 2wks pi, 4wks pi, and 8wks pi mice (n = 6 independent animals per group). (A) Integrated 1-100 Hz broadband power with individual animals overlaid; horizontal bars show means and error bars show SD. The four-group comparison was significant (Kruskal-Wallis H = 14.85, p = 0.00195, FDR q = 0.00439). The Control-versus-4wks pi contrast was significant after FDR correction (Welch's t = 11.48, q = 7.28 x 10-6, Hedges' g = -6.12). (B) Group-mean change relative to Control for delta, theta, alpha, beta, low-gamma, and high-gamma bands. Four-group FDR q values are shown below the heat map; low gamma did not reach the FDR threshold (q = 0.0569), whereas all other bands did (q <= 0.0409).

Supplementary Figure S2.

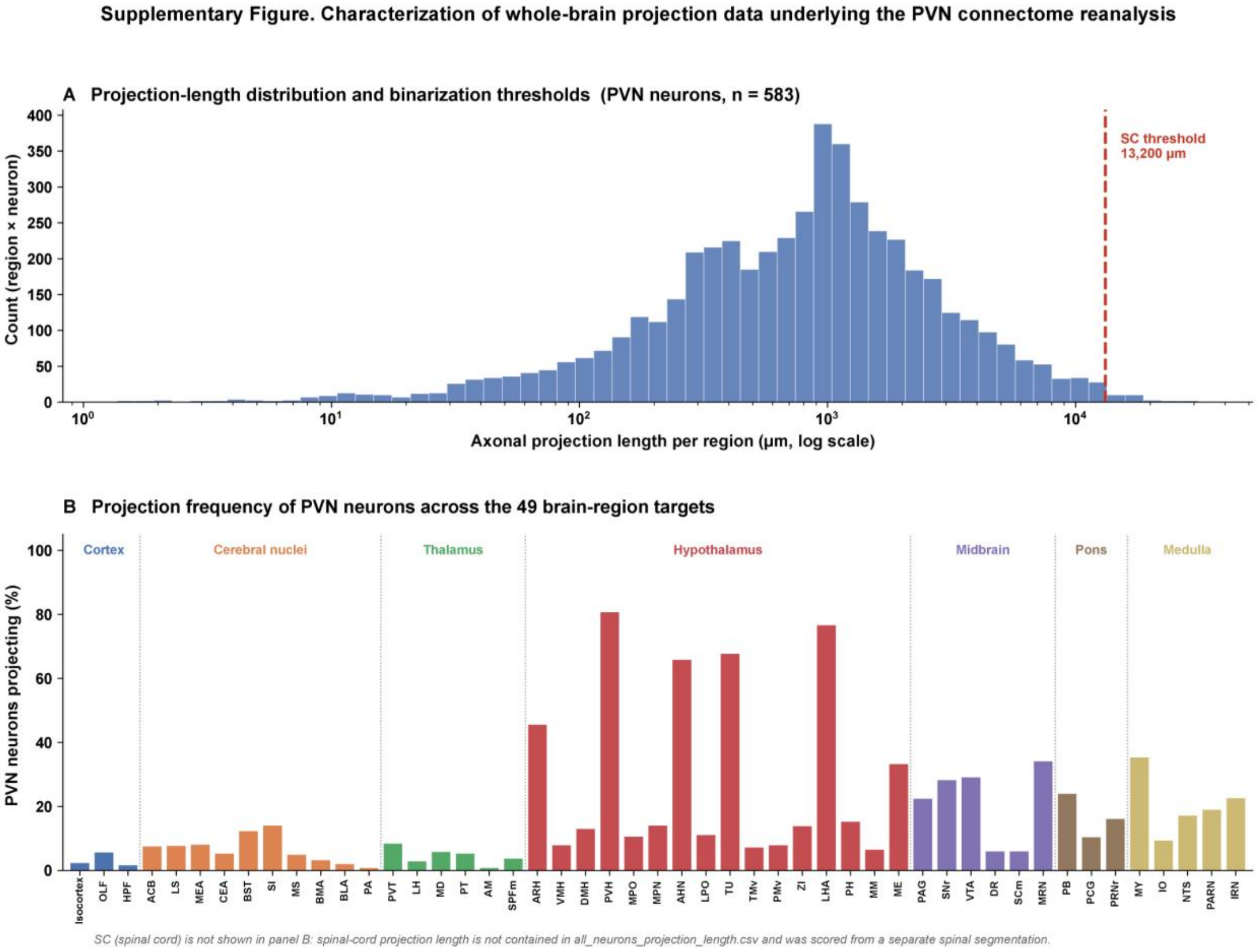

Supplementary Figure S2. Characterization of the whole-brain projection data underlying the PVN single-neuron connectome reanalysis. Related to Fig. 2I–L. Analysis is based on the 583 neurons with paraventricular hypothalamic nucleus (PVH/PVHd) soma location in the source projectome dataset (Jiao et al., 2025), of which 574 were retained for clustering after quality control. (A) Distribution of reconstructed axonal projection lengths, pooled across the 49 brain-region targets (log-scaled x-axis; each count is one region × neuron). The dashed red line marks the 13,200 µm threshold used to score spinal-cord projection. Brain-region projection was binarized at any reconstructed length > 0 µm; under this criterion 7.8% of scored brain-region projections are shorter than 100 µm and 99.4% fall below the spinal-cord threshold, indicating that the two binarization criteria operate on markedly different length scales. (B) Percentage of PVN neurons with a non-zero reconstructed projection to each of the 49 brain-region targets, ordered and color-coded by the eight anatomical divisions. Spinal cord is not shown in (B): spinal-cord projection length is not contained in the projectome length table and was scored from a separate spinal segmentation.

Supplementary Figure S3.

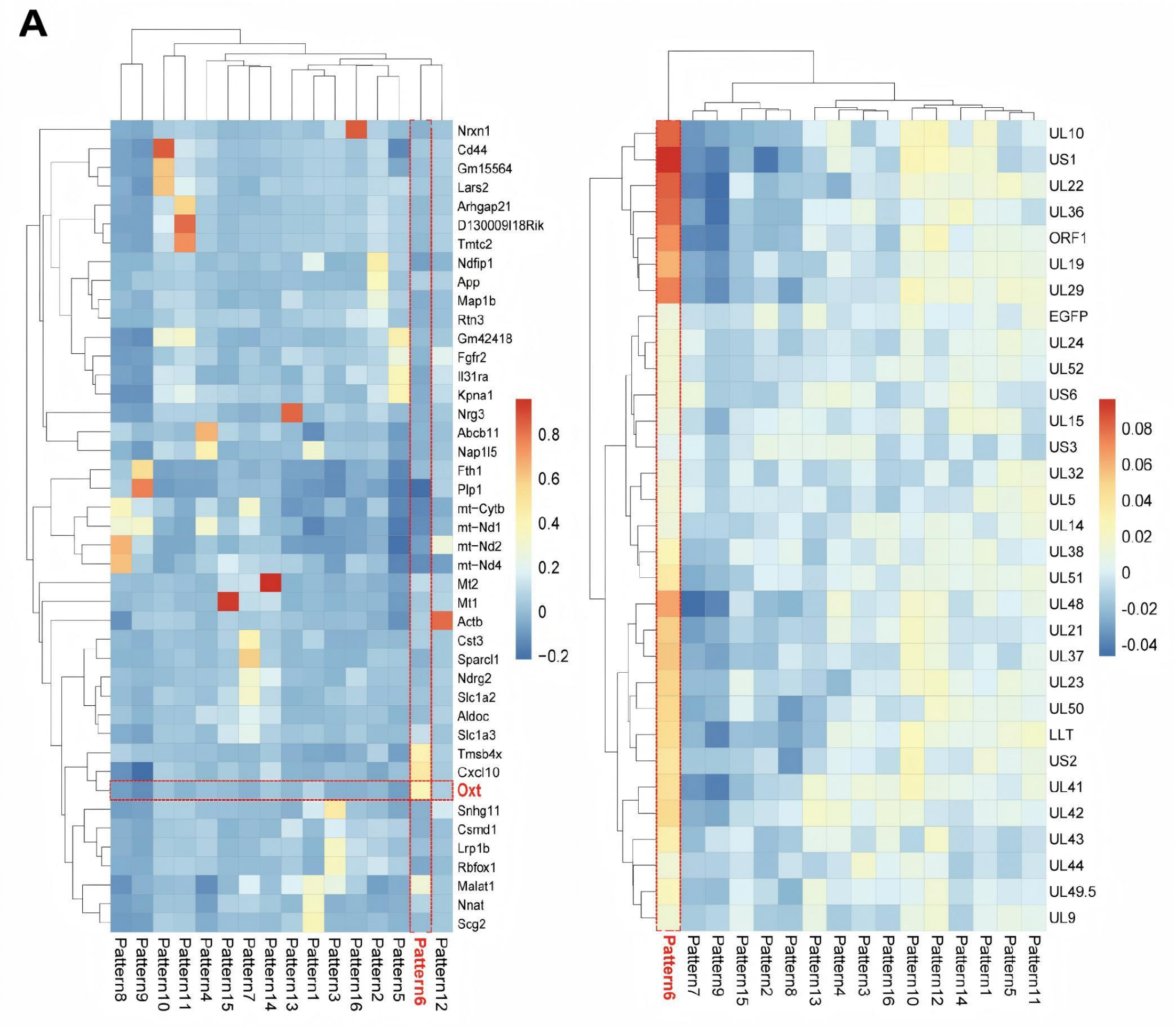

Supplementary Figure S3. Pattern 6 identifies coordinated enrichment of Oxt and pseudorabies virus transcripts in the mPVN spatial-transcriptomic dataset. Related to Fig. 3F-I. (A) Hierarchically clustered heat map of pattern-associated scores for host genes across SpaGene co-expression modules. Pattern 6 is highlighted by the red dashed box; the Oxt row and Pattern 6 column show concordant enrichment. (B) Corresponding heat map of PRV-gene pattern-associated scores. Pattern 6, outlined in red, shows broad enrichment across PRV transcripts. Color scales denote normalized pattern-associated scores. This exploratory analysis was performed on the same mPVN Stereo-seq section shown in Fig. 3E-I and does not represent an independent biological replicate.

Supplementary Figure S4.

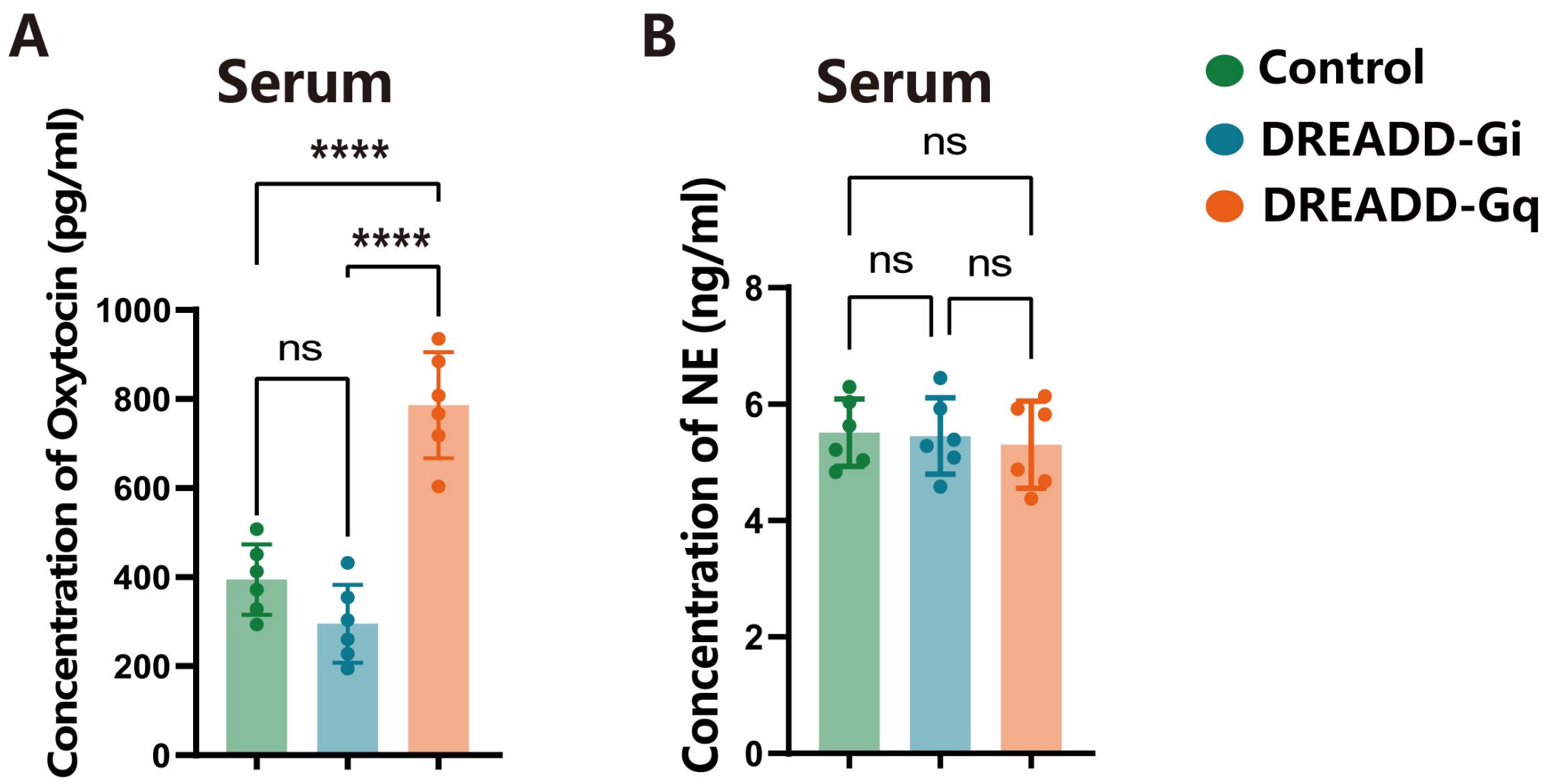

Supplementary Figure S4. Serum oxytocin and norepinephrine concentrations following chemogenetic manipulation of PVNOXT neurons. Related to Fig. 4D. (A) Serum oxytocin and (B) serum norepinephrine (NE) were measured at 4wks pi in Control mice and mice subjected to chemogenetic inhibition (DREADD-Gi; hM4D(Gi)) or chemogenetic activation (DREADD-Gq; hM3D(Gq)) of PVNOXT neurons. Serum oxytocin was significantly higher in DREADD-Gq than in Control and DREADD-Gi, whereas DREADD-Gi did not differ from Control. Serum NE did not differ among groups. n = 6 mice per group; each dot represents one mouse. One-way ANOVA followed by Tukey's multiple-comparison test. ****$p < 0.0001$; ns, not significant.

Supplementary Figure S5.

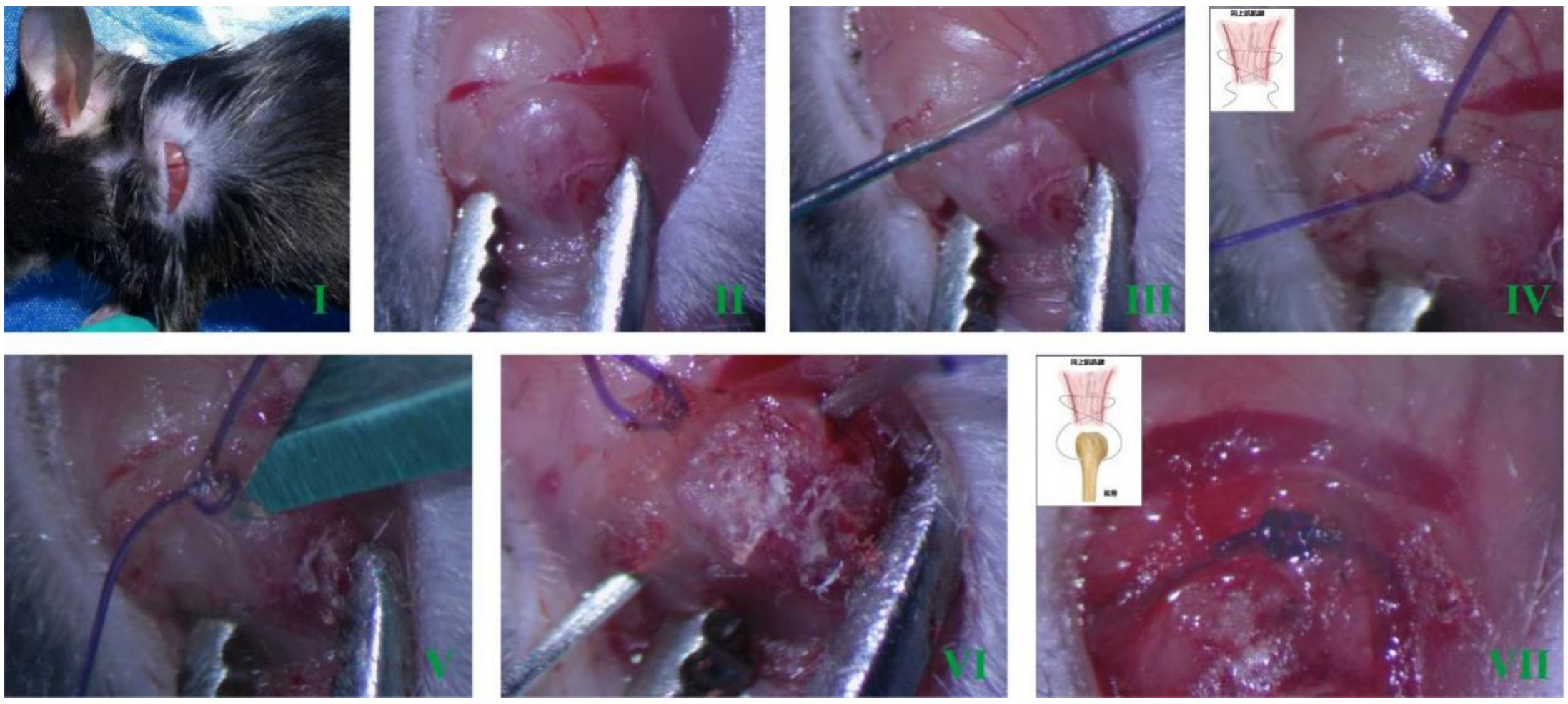

Supplementary Figure S5. Surgical Procedure: I. Place the mouse in right lateral decubitus position. Internally rotate the left shoulder joint and make a longitudinal incision approximately 1 cm in length. II. Transect the deltoid muscle below the transverse blood vessels to expose the supraspinatus muscle insertion. III. Elevate the supraspinatus tendon to fully expose the tendon and its insertion site. IV. Perform figure-of-eight crossover suture on the supraspinatus tendon using 6-0 double-needle PDS sutures. V. Sever the supraspinatus tendon from its humeral insertion using a surgical blade, then carefully scrape the fibrocartilage at the tendon-bone interface insertion site down to the subchondral bone layer. VI. Create oblique downward bone tunnels bilaterally along the humeral insertion of the supraspinatus tendon to form a figure-of-eight crossover bone tunnel configuration. VII. Pass the PDS sutures through the crossover bone tunnels, tighten the sutures, and secure with knots.
